# A *trans*-acting Variant within the Transcription Factor RIM101 Interacts with Genetic Background to Determine its Regulatory Capacity

**DOI:** 10.1371/journal.pgen.1005746

**Published:** 2016-01-11

**Authors:** Timothy Read, Phillip A. Richmond, Robin D. Dowell

**Affiliations:** 1 Department of Molecular, Cellular and Developmental Biology, University of Colorado, Boulder, Boulder, Colorado, United States of America; 2 BioFrontiers Institute, University of Colorado, Boulder, Boulder, Colorado, United States of America; Washington University School of Medicine, UNITED STATES

## Abstract

Most genetic variants associated with disease occur within regulatory regions of the genome, underscoring the importance of defining the mechanisms underlying differences in regulation of gene expression between individuals. We discovered a pair of co-regulated, divergently oriented transcripts, *AQY2* and *ncFRE6*, that are expressed in one strain of *Saccharomyces cerevisiae*, ∑1278b, but not in another, S288c. By combining classical genetics techniques with high-throughput sequencing, we identified a *trans*-acting single nucleotide polymorphism within the transcription factor *RIM101* that causes the background-dependent expression of both transcripts. Subsequent RNA-seq experiments revealed that *RIM101* regulates many more targets in S288c than in ∑1278b and that deletion of *RIM101* in both backgrounds abrogates the majority of differential expression between the strains. Strikingly, only three transcripts undergo a significant change in expression after swapping *RIM101* alleles between backgrounds, implying that the differences in the *RIM101* allele lead to a remarkably focused transcriptional response. However, hundreds of *RIM101*-dependent targets undergo a subtle but consistent shift in expression in the S288c *RIM101*-swapped strain, but not its ∑1278b counterpart. We conclude that ∑1278b may harbor a variant(s) that buffers against widespread transcriptional dysregulation upon introduction of a non-native *RIM101* allele, emphasizing the importance of accounting for genetic background when assessing the impact of a regulatory variant.

## Author Summary

Large-scale genome profiling studies have revealed that disease-associated variants occur most frequently within regulatory regions of DNA. In order to connect variants (genotypes) to expression profiles (phenotypes), it is necessary to have a thorough understanding of the mechanisms underlying transcriptional regulation across genetic backgrounds. In this study we used a high-throughput method for identification of the genetic variant responsible for differential expression of a pair of divergently oriented transcripts between two strains of *S*. *cerevisiae*. We discovered a single nucleotide polymorphism within the transcription factor, *RIM101*, that is required for co-regulation of both transcripts. Furthermore, we showed that the expression pattern of the divergently oriented transcripts was determined by the SNP across a diverse array of genetic backgrounds. The extent to which the SNP affects genome-wide transcriptional profiles was strongly influenced by the genetic background, a phenomenon that likely contributes to the astounding phenotypic diversity observed within a population.

## Introduction

Incorporating genomic information into clinical practice is a major focus of personalized medicine. Despite the discovery of a large number of disease-associated genetic variants [1,2], few clinical treatments have been developed that take into account such information [3]. Furthermore, most disease-associated variants occur within regulatory regions of DNA [4,5], making it particularly difficult to predict the biological processes they affect. Determining the mechanisms by which variants influence regulation, and hence phenotypic diversity among individuals, is paramount to a thorough understanding of functional genomics.

Uncovering the biological mechanisms underlying regulatory variants, as well as how variants interact with the myriad of genetic backgrounds present within a population, is a major focus of current research. It has become increasingly clear that genetic background contributes to phenotypes [6–11] resulting in the seemingly infinite diversity observed in nature, even within an individual species. Recent studies suggest that transcript levels can be both powerful readouts for and determinants of disease states [12–15]. However, similar to other cellular phenotypes, expression differences among individuals are the product of an exceedingly complex genetic landscape. One reason for this complexity is that even subtle mutations in transcription factors can impart distinct regulatory roles when present within alternative genetic contexts [16,17].

Strategies linking genetic variants to regulation of gene expression often consist of one of two approaches. Expression quantitative trait loci (eQTL) studies combine genome-wide expression data with genome sequence information to uncover expression-influencing variants, including those linked to disease [18–24]. In addition to variants themselves, eQTL studies have uncovered many important genetic principles. For example, expression-influencing variants that occur near the gene being regulated, or in *cis*, tend to influence a single gene, whereas variants distal to the gene being regulated, or in *trans*, typically influence expression of many loci [25–29]. In contrast to the genome-wide eQTL approach, which correlates genetic variants with expression differences, much of our knowledge of the mechanisms underlying gene expression regulation comes from detailed, single-locus studies [30–34]. However, such studies usually do not consider the effects of naturally occurring genetic variation on gene expression.

We sought to combine attributes of genome-wide and single-locus studies to understand the mechanistic basis by which genetic variants result in altered regulation of gene expression between two strains of *Saccharomyces cerevisiae*. Here we describe principles underlying the complexity of gene expression regulation and report evidence that genetic background strongly influences the extent to which a variant affects transcript levels throughout the genome.

## Results

### *Cis* variation controls background-specific co-regulation of *AQY2* and *ncFRE6*

Two strains of *S*. *cerevisiae*, S288c and ∑1278b, are a model system for understanding how intraspecies genome sequence variation impacts phenotype [35]. We initially sought to identify a locus where we could directly test how naturally occurring genetic variation impacts transcription factor (TF) binding and associated transcript levels. Ideally, the locus would harbor at least one SNP within a predicted TF binding site, allowing us to directly test the effect of the SNP on TF binding. If TF binding is modulated by the SNP, we could subsequently determine its influence on nearby transcript levels, effectively defining a mechanism by which genome sequence influences expression in *cis*. Specifically, we aimed to identify a genomic region that displays strain-specific binding of a TF that correlates with a nearby strain-specific expression difference.

In order to correlate TF binding with gene expression, we first performed strand-specific RNA-seq on S288c and Σ1278b. Not surprisingly given the high degree of sequence similarity (99.7%) between the strains, the majority of transcripts are expressed at similar levels between the strains **(S1A Fig).** However, about 20% of genes are differentially expressed (DESeq, n = 1207, Padj≤0.0005, minimum average expression ≥ 100 reads) **(S1B Fig and S4 Table).** Of the differentially expressed genes, gene ontology (GO) terms are enriched for categories such as transcription factor activity, mRNA binding, and oxidoreductase activity (Pval≤0.002) **(S2 Table)**. In addition to protein-coding genes, we identified 82 differentially expressed antisense transcripts (DESeq, n = 82, Padj≤0.0005, minimum average expression ≥ 50 reads) **(S1C Fig and S5 Table).**

To study how expression patterns are gained or lost throughout intraspecies evolution, we initially focused on loci displaying an extreme differential expression phenotype between the strains (i.e. on in one strain and off in the other) **(n = 62, S1 Table)**. To identify a potentially regulatory SNP involved in the birth or death of a transcript, we examined the promoter regions of all 62 “extreme expressors.” Roughly 25% of extreme expressors harbor a SNP within 50 basepairs of their transcription start site. In one case, a SNP is located very near the transcription start site of a non-coding RNA, *ncFRE6*, which is transcribed in an antisense orientation to the *FRE6* ORF in ∑1278b, but not in S288c (S2B **Fig**). Closer inspection of the DNA sequence surrounding the *ncFRE6*-associated SNP revealed that a consensus *Reb1* binding motif is interrupted in S288c relative to ∑1278b **(S2B Fig, Red line)**. We hypothesized that *Reb1* binding activates *ncFRE6* expression in ∑1278b relative to S288c.

In parallel with RNA-seq, we monitored occupancy of *Reb1* in S288c and ∑1278b by ChIP-seq (**S1 Text**). Because *REB1* shares 100% sequence identity between S288c and ∑1278b it is not surprising that most *Reb1* binding events are conserved between the backgrounds (83% of total binding events) (**S2A Fig**). However, there are a small number of strain-unique *Reb1* binding events, including one at the position of the *ncFRE6*-associated SNP in ∑1278b and not in S288c. Because the SNP disrupts a preferred *Reb1* binding site in S288c relative to ∑1278b we interconverted the SNP between backgrounds in an attempt to rescue binding in S288c and/or abolish binding in ∑1278b. We used the “delitto perfetto” method [36] to interconvert the SNP and observed that it is indeed necessary and sufficient to cause the *Reb1* binding discrepancy between the strains (**S2C Fig**). However, we were surprised to observe that abolishing *Reb1* binding in ∑1278b did not reduce expression of *ncFRE6*, and rescuing binding in S288c did not increase expression of *ncFRE6* in S288c (**S2D Fig**). This result highlights the importance of single locus studies for uncovering the causal variant(s) driving differences in transcript levels rather than assuming that correlations between TF binding and transcript levels are meaningful.

**Fig 1:**
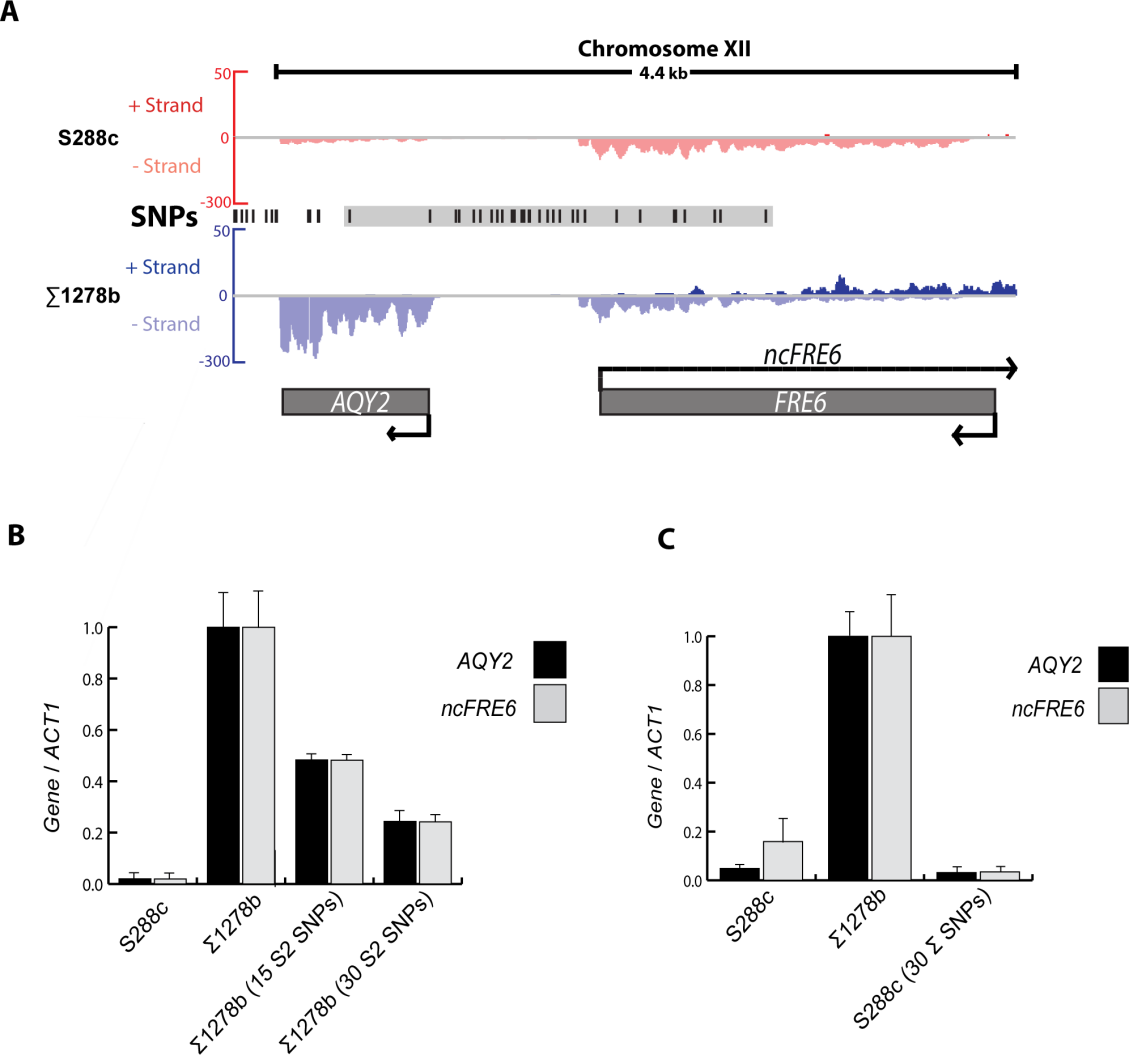
*Cis* variation controls background-specific co-regulation of *AQY2* and *ncFRE6*. (A) Integrated Genome Viewer (IGV) screenshot displaying strand–specific RNA-seq of the *AQY2/ncFRE6* region in S288c (Red) and ∑1278b (Blue). S288c chromosomal coordinates ChrXII: 35,200–39,570. Data are displayed as positive (Watson) strand above the axis and negative (Crick) strand below the axis. Single nucleotide variations between the strains are shown in black. The grey box highlights 30 interconverted SNPs. (B) Relative expression of *AQY2* (black) and *ncFRE6* (grey) (Color scheme maintained throughout results) measured by qRT-PCR. (C) Relative expression of *AQY2* and *ncFRE6* measured by qRT-PCR. (All qRT-PCR data displayed as mean and error bars represent SEM of two or three biological replicates throughout results, unless otherwise noted.) Data were normalized to *ACT1* levels. ∑1278b wildtype (wt) levels for both *AQY2* and *ncFRE6* were normalized to one throughout the manuscript. *AQY2* and *ncFRE6* levels in all other strains are displayed relative to ∑1278b levels. RNA was prepared independently for each qRT-PCR experiment.

Since differential *Reb1* binding does not cause the differential expression of *ncFRE6* between S288c and ∑1278b, we asked whether other *cis* elements influenced expression. *AQY2* is a divergently oriented gene that originates ~1kb upstream of the *ncFRE6* transcription start site and is also expressed specifically in ∑1278b. *AQY2* encodes a water channel that is disrupted by a premature stop codon in the vast majority of sequenced strains of *S*. *cerevisiae*, including S288c, but is functional in ∑1278b [37]. The *AQY2/ncFRE6* promoter region has undergone significant genetic drift between S288c and ∑1278b. Harboring 21 SNPs, the *AQY2/ncFRE6* intergenic region is one of the most sequence-variable promoters between S288c and ∑1278b (**S3 Fig**). Because a large number of SNPs within the region, both intergenic and within the body of each transcript, disrupt potential TF binding sites **(Fig 1A, Grey box, and S3 Table)**, we hypothesized that one or more of the SNPs drive the differential expression of *AQY2* and/or *ncFRE6*. Indeed, replacing all 30 SNPs in ∑1278b with those from S288c results in ~75% reduction of both *AQY2* and *ncFRE6* and replacing only the 15 *AQY2*-proximal SNPs results in ~50% reduction in the transcripts, indicating that DNA elements in both halves of the intergenic region contribute to expression levels of both *AQY2* and *ncFRE6* in Σ1278b **(Fig 1B)**. Surprisingly, the expression levels of both transcripts were reduced by nearly identical magnitudes in ∑1278b promoter-altered strains, implying that the two divergently oriented transcripts are co-regulated in *cis*. However, while introducing the S288c *cis* context into Σ1278b dramatically reduced expression of the transcripts, incorporation of 30 ∑1278b SNPs into S288c was completely ineffective at increasing *AQY2* and/or *ncFRE6* transcript levels **(Fig 1C)**. Taken together, these results indicate that *AQY2* and *ncFRE6* are likely co-regulated and that a *trans*-acting factor(s) ultimately determines whether *AQY2* and/or *ncFRE6* are expressed.

### A single *trans* factor is epistatic to *cis*-linked variation with regards to expression of *AQY2* and *ncFRE6*

**Fig 2:**
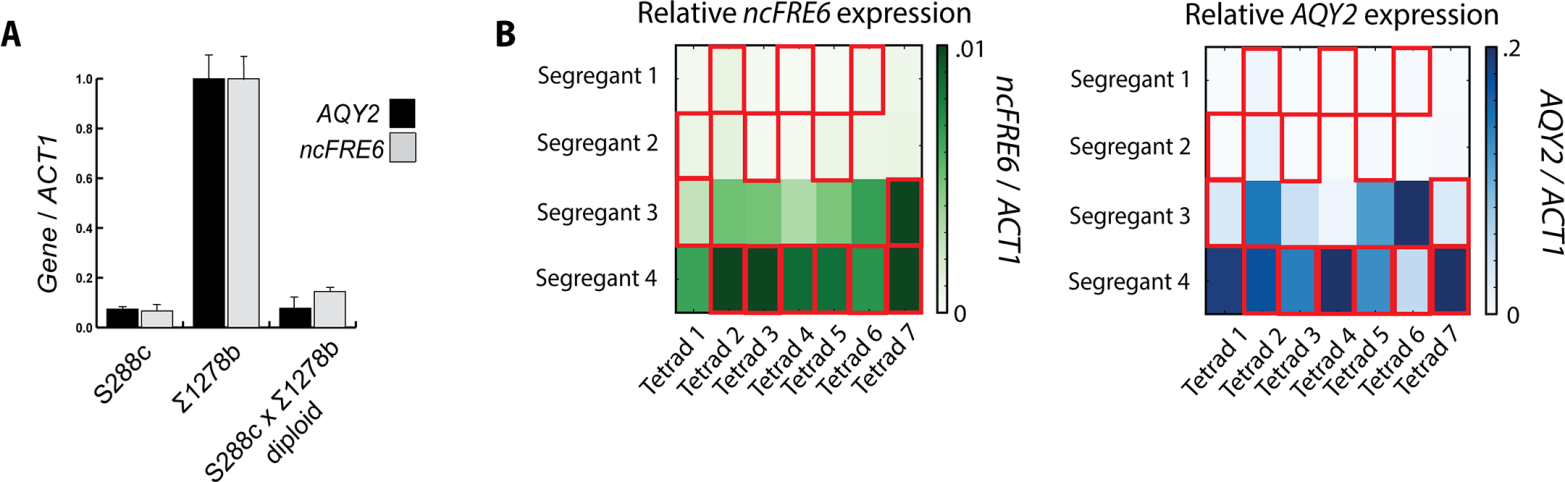
A single *trans* factor is epistatic to *cis*-linked variation with regards to expression of *AQY2* and *ncFRE6*. (A) Relative expression of *AQY2* and *ncFRE6* in S288c and ∑1278b wildtype haploid strains and an S288c X ∑1278b diploid strain measured by qRT-PCR. (B) Heatmap displaying relative expression of *AQY2* (Blue) and *ncFRE6* (green) in 28 segregants of an S288c X ∑1278b heterozygous diploid. Segregants were numbered according to expression level of *ncFRE6* (segregants 1 and 2 are non-expressing strains and 3 and 4 are expressing strains) for each of seven tetrads dissected.

To learn about the genetic nature of the *trans* factor(s) that causes differential regulation of *AQY2* and *ncFRE6* between S288c and ∑1278b, we crossed the two strains and monitored expression of the transcripts in a heterozygous diploid. Neither *AQY2* nor *ncFRE6* expression is observed in the diploid strain **(Fig 2A)**, implying that the S288c expression phenotype is dominant. To determine whether one or more *trans* factors control expression of *AQY2* and/or *ncFRE6*, we performed tetrad analysis assaying for expression of both *AQY2* and *ncFRE6*. For each tetrad analyzed **[38]**, two haploid segregants express both *AQY2* and *ncFRE6* concurrently while the other two express neither transcript **(Fig 2B)**. This 2:2 pattern of inheritance suggests that a single *trans* factor controls the on/off state of both transcripts, and that co-expression of the transcripts is conserved even in the unique genetic admixtures of the segregants.

We Sanger sequenced the *AQY2/ncFRE6 cis* context within each haploid segregant to determine whether the S288c *cis* context exhibits a similar level of promoter activity as the ∑1278b *cis* context in terms of expression of *AQY2/ncFRE6* (**Fig 2B, Red boxes harbor S288c *AQY2/ncFRE6 cis* context**). Indeed, both the S288c and Σ1278b *cis* contexts permit expression of *AQY2/ncFRE6*, but only in the absence of the *trans* factor. Furthermore, expression levels varied considerably between *AQY2/ncFRE6*-expressing segregants. Contrary to the results of the promoter swapped ∑1278b strain (**Fig 1B**), the segregants that harbor the S288c *cis* context tend to express *higher* levels of *AQY2/ncFRE6* than those harboring the ∑1278b *cis* context (**Fig 2B and 2C**). This result implies that there are additional factors that alter the expression levels of *AQY2/ncFRE6*, but only within genetic backgrounds that lack the epistatic *trans*-factor. Furthermore, given that *aqy2* codes for a non-functional protein in S288c, it is somewhat surprising that the S288c *cis* context possesses robust promoter activity.

### *RIM101* controls expression of both *AQY2* and *ncFRE6* in *trans*

**Fig 3:**
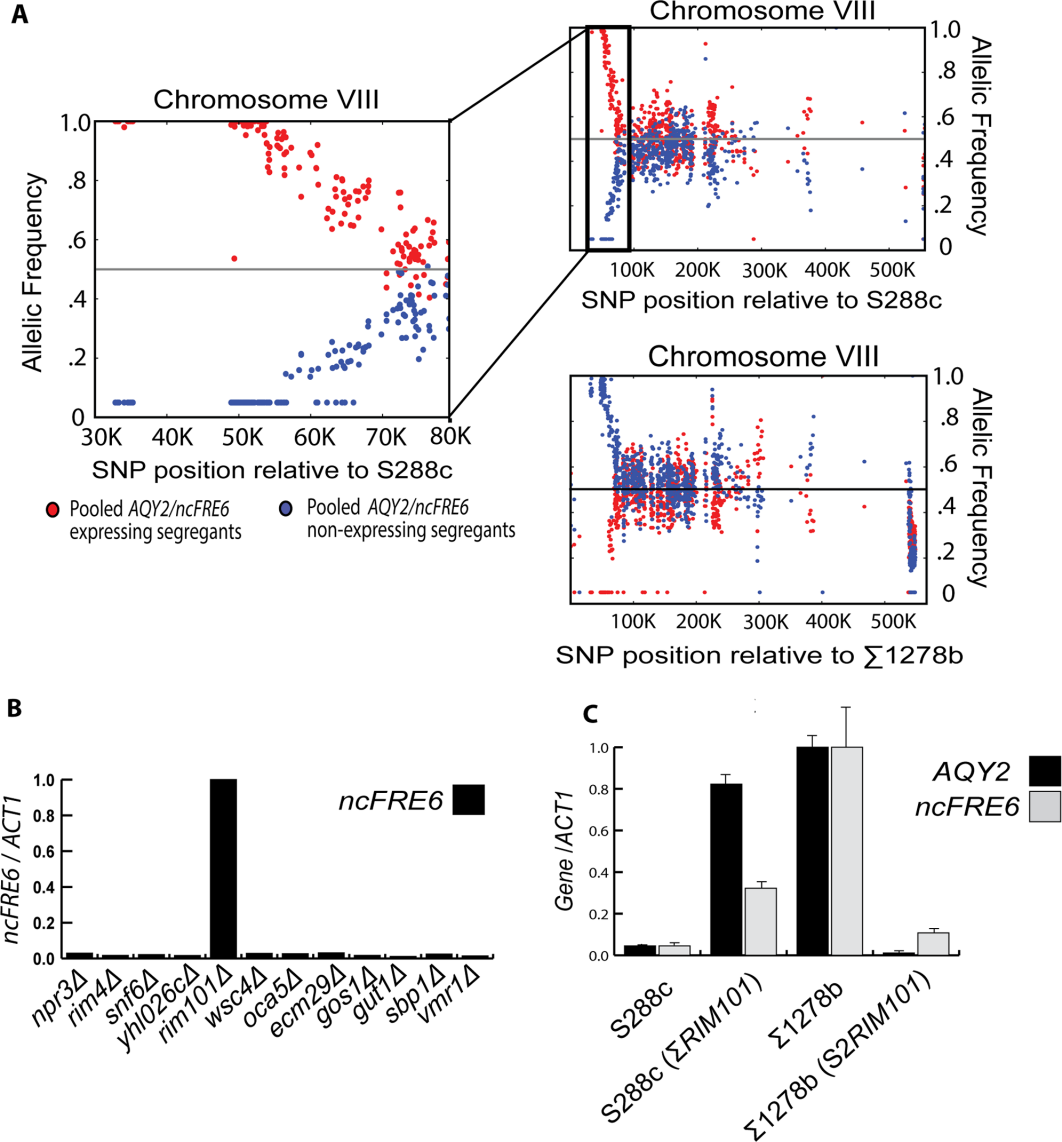
*RIM101* controls expression of both *AQY2* and *ncFRE6* in *trans*. (A) Scatter plot displaying the allelic frequency of every SNP (dots) between S288c and ∑1278b in 14 *AQY2/ ncFRE6* expressing strains (segregants 3 and 4 from Fig 2, red dots) and 14 non-expressing strains (segregants 1 and 2 from Fig 2, blue dots) across chromosome VIII (all chromosomes displayed in S5 Fig). Strains were pooled based on expression of *AQY2/ncFRE6*, sequenced, and mapped to each reference genome. X-axis represents position along chromosome VIII. Y-axis represents the allelic frequency of each SNP relative to the genome being mapped to for each pool. Zoomed region represents the distal left arm of chromosome VIII where all SNPs segregate with either the expressing or non-expressing strains. (B) qRT-PCR measuring *ncFRE6* in gene deletions within the 35kb region identified in Fig 3A. (C) qRT-PCR measuring *AQY2* and *ncFRE6* in wildtype and *RIM101*-interconverted strains.

In order to map the genetic location of the *trans* factor that causes differential expression of *AQY2/ncFRE6* between S288c and Σ1278b, we combined bulked segregant analysis [39] with high throughput sequencing. A similar approach was developed previously using microarrays to map complex phenotypes influenced by a large number of loci [40]. We reasoned that the single dominant repressor would be present in all of the non-expressing segregants of an S288c x ∑1278b cross. Therefore, the variant driving differential expression of *AQY2/ncFRE6* should always segregate according to the repression phenotype **(S4 Fig)**. We isolated genomic DNA from 28 segregants: 14 that express *AQY2* and *ncFRE6* and 14 that do not. We pooled equal amounts of DNA from each strain in the two sets and performed high throughput sequencing of the pools. We then sought to identify regions of the genome inherited exclusively from S288c in the non-expressing strains and from Σ1278b in the expressing strains. Only one region fit this criteria: an approximately 35kb region near the left arm of chromosome VIII **(S1 Text and Figs 3A and S5)**. Because the heterozygous diploid does not express *AQY2* or *ncFRE6*, we reasoned that S288c likely harbors a repressor within this region.

To identify the locus within this region responsible for repression of *AQY2/ncFRE6* in S288c, we screened the S288c deletion library [41] for expression of *ncFRE6* in each of 12 gene deletions within the 35kb region. Of the deletions tested, only one, *rim101Δ*, de-repressed the transcripts **(Fig 3B)**, strongly suggesting that the *RIM101* allele harbors the *trans* factor that represses *AQY2/ncFRE6* in S288c. In support of this hypothesis we note that *RIM101* is a well-characterized zinc finger transcriptional repressor and is one of the most sequence-variable transcription factors between S288c and ∑1278b, harboring 18 SNPs, 13 of which are non-synonymous **(S6 Fig and S1 Text)**.

To confirm that the polymorphic *RIM101* allele controls expression of *AQY2/ncFRE6*, we interconverted the entire *RIM101* open reading frame (S288c: ChrVIII 51111–52988, Σ1278b: ChrVIII 49766–51655) between the strains and measured expression of *AQY2/ncFRE6*. Interconverting the *RIM101* allele is sufficient to repress expression in Σ1278b and to rescue expression in S288c, confirming that the *RIM101* alleles confer distinct *trans*-acting regulatory capacity with regards to *AQY2/ncFRE6* expression **(Fig 3C)**. We concluded that one or more of the sequence variations between the strains is responsible for the difference in *RIM101* activity.

*RIM101* is known to contribute to several phenotypes, including being required for haploid invasive growth in ∑1278b [42]. S288c cannot invade agar due to a loss of function mutation in the TF *FLO8* and therefore is insensitive to null mutations in *RIM101*. We reasoned that the differences within the S288c and ∑1278b *RIM101* allele could affect the invasive growth phenotype [43] in ∑1278b. However, the ∑1278b strain harboring the S288c *RIM101* allele, ∑1278b(S2*RIM101*), did not lose the ability to invade agar, implying that differences between the S288c and ∑1278b *RIM101* alleles do not affect the invasive growth phenotype (**S7 Fig**).

### Most differential expression between S288c and Σ1278b is *RIM101*-linked

**Fig 4:**
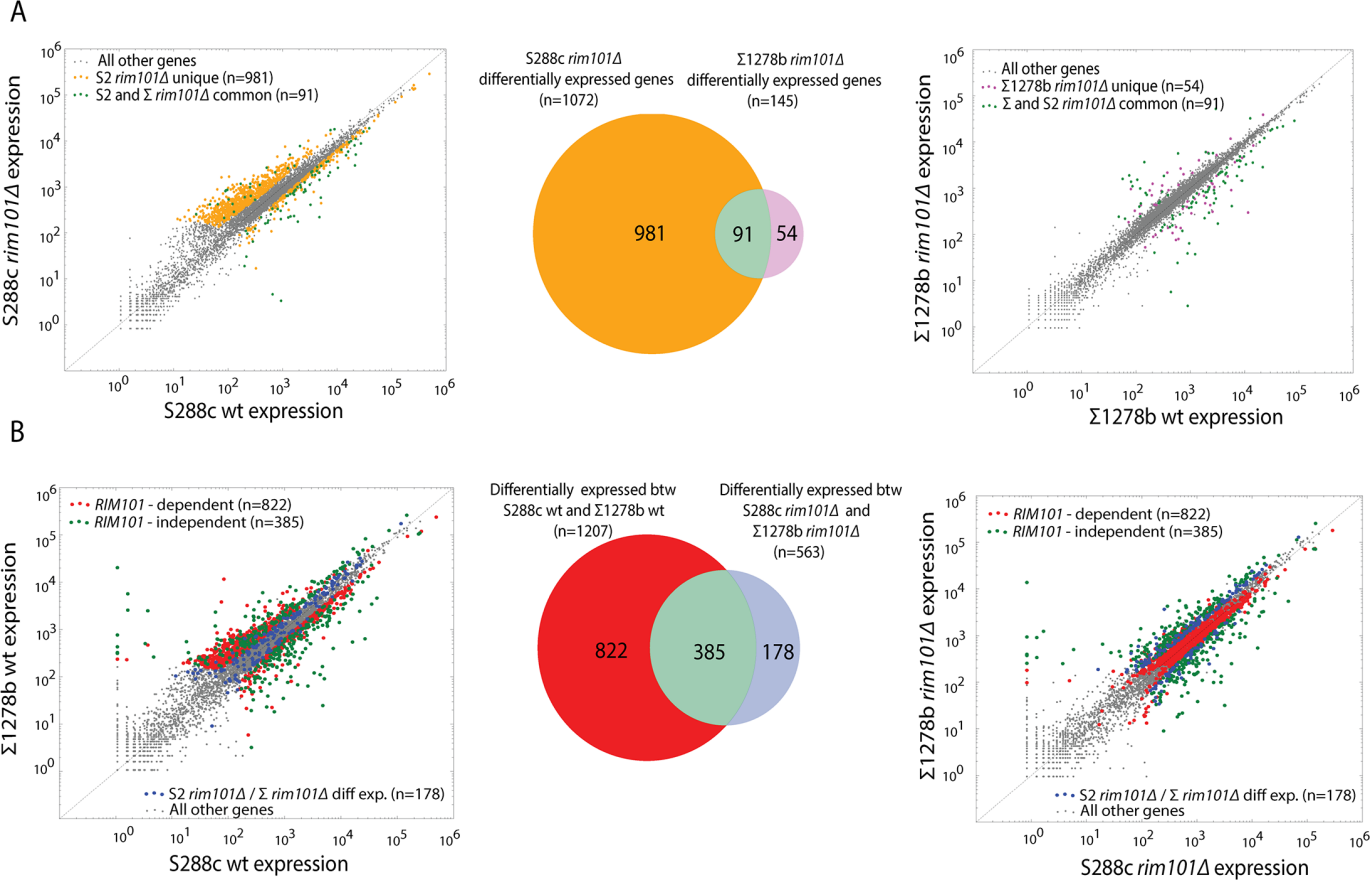
Most differential expression between S288c and Σ1278b is *RIM101*-linked. (A) Scatter plots displaying expression levels of each gene (dots) in *rim101∆* strains relative to wildtype as measured by RNA-seq. Venn diagram represents the number of genes differentially expressed in S288c *rim101∆* relative to S288c wt (orange) or between ∑1278b *rim101∆* and ∑1278b wt (magenta). The overlap (green) represents genes differentially expressed in both S288c *rim101∆* and ∑1278b *rim101∆* strains relative to respective wildtype strains. Dots on scatter plots are colored according to the Venn diagram. (B) Scatter plots displaying expression levels of each gene (dots) between *RIM101* wt S288c and ∑1278b strains (left) or between S288c *rim101∆* and ∑1278b *rim101∆* strains (right). Venn diagram represents the number of genes differentially expressed between S288c and ∑1278b wt strains (Red) or between S288c *rim101∆* and ∑1278b *rim101∆* strains (blue). The overlap (green) represents genes differentially expressed in both comparisons. Dots on scatter plots are colored according to the Venn diagram. RNA-seq performed on 2 biological replicates for each strain. Differential expression called by DESeq with Padj = 0.0005.

To assess the impact of *RIM101* on genome-wide expression, we performed RNA-seq on *RIM101* deletion strains in S288c and ∑1278b. Consistent with *RIM101*’s role as a transcriptional repressor, the majority of the genes whose expression level changes upon deletion of *RIM101* in S288c became de-repressed (771 upregulated, 301 downregulated) **(S4 Table)**. Surprisingly, the effect of deleting *RIM101* in S288c was much larger than in Σ1278b **(Fig 4A)**. While 1072 genes change expression levels in S288c *rim101∆* relative to S288c wildtype, only 145 change in ∑1278b *rim101∆* relative to ∑1278b wildtype. Furthermore, the ratio of de-repressed to repressed genes is opposite in the Σ1278b *RIM101* deletion (45 upregulated, 100 downregulated). This result suggests that *RIM101* is a stronger repressor in S288c than in ∑1278b. Consistent with a loss of repressive capacity in ∑1278b relative to S288c, we note that *AQY2/ncFRE6* levels do not change in the ∑1278b *rim101∆* strain. Nevertheless, 145 genes do change expression in ∑1278b *rim101∆*, implying that the disparate response to deletion of *RIM101* is not due to a complete loss of function of the ∑1278b *RIM101* allele.

We next sought to determine the extent to which genome-wide differential expression between S288c and ∑1278b can be attributed to *RIM101*. We reasoned that genes that are differentially expressed between the wildtype strains but not the *RIM101* deletion strains are *RIM101-*dependent because removal of *RIM101* from the system eliminates the observed interstrain differential expression. Hence, these differences in expression level between S288c and ∑1278b can be attributed to differences in *RIM101*-mediated regulation. Surprisingly, of 1207 differentially expressed genes between S288c and Σ1278b, over two-thirds (822) are in some way dependent on the presence of *RIM101* **(Fig 4B, Red)**.

We next asked how expression of the 822 *RIM101*-dependent transcripts (as defined in Fig 4B) changes upon loss of the *RIM101* allele in each background. Deleting *RIM101* in S288c results in a shift in *RIM101-*dependent gene expression toward ∑1278b wildtype levels **(S8A Fig)**. However, deletion of *RIM101* in ∑1278b did not result in a shift toward S288c wildtype levels **(S8B Fig)**. This asymmetric response to *RIM101* deletion is consistent with *RIM101* possessing augmented repressive capacity in S288c relative to ∑1278b.

### The *RIM101* allele achieves remarkable specificity, but genetic background controls its regulatory capacity

**Fig 5:**
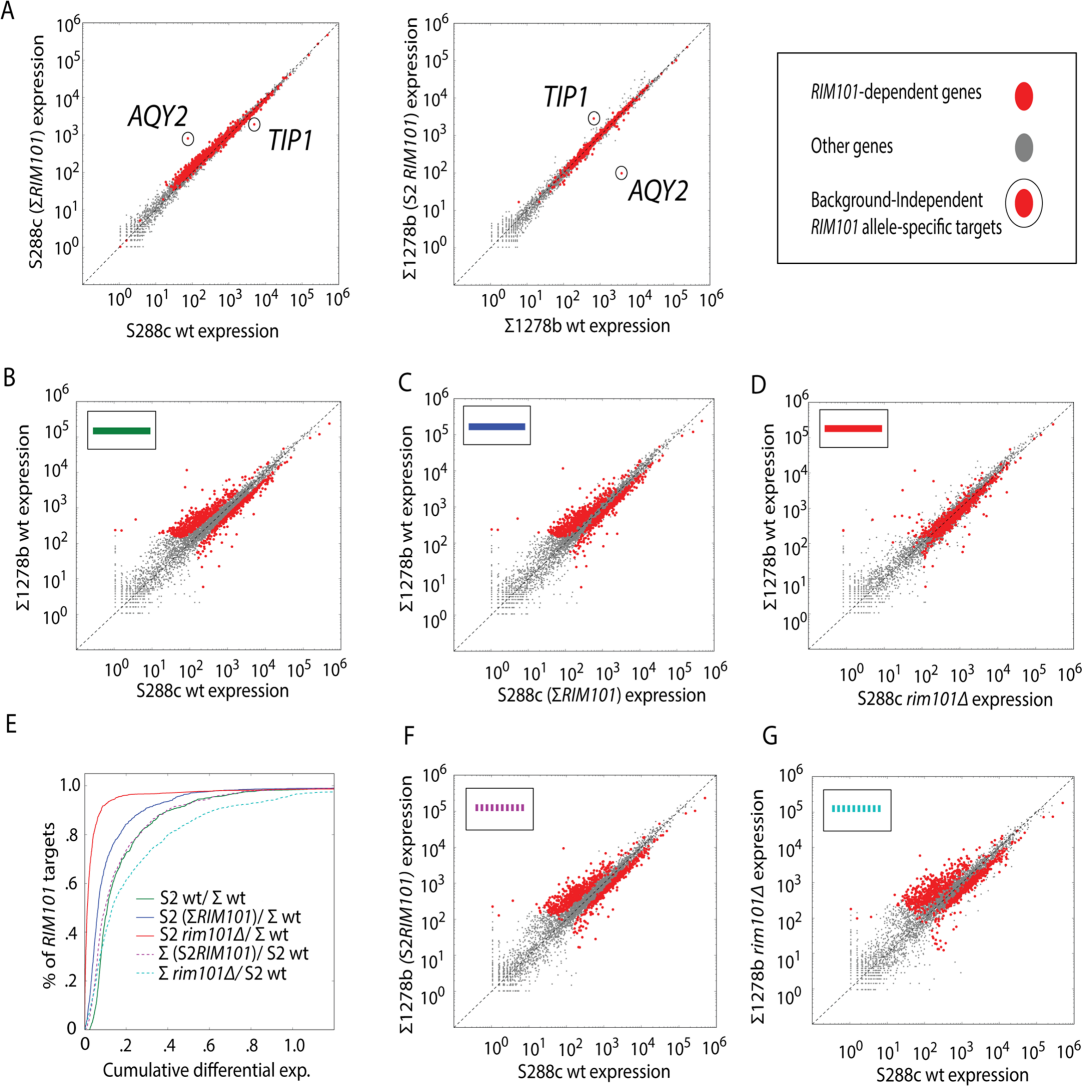
The *RIM101* allele achieves remarkable specificity, but genetic background controls its regulatory capacity. (A) Scatter plots displaying expression levels for each gene (dots) in *RIM101* interconverted strains relative to their respective wildtype strains (Red = *RIM101*-dependent genes as defined in Fig 4). (B) Scatter plot displaying levels of each gene in Σ1278b relative to S288c wildtype strains (Red = *RIM101*-dependent genes). (C) Scatter plot displaying expression levels in the *RIM101*-interconverted strain, S288c(Σ*RIM101*) relative to Σ1278b wildtype (Red = *RIM101*-dependent genes). (D) Scatter plot displaying expression levels in S288c *rim101Δ* relative to Σ1278b wildtype (Red = *RIM101*-dependent genes). (E) Cumulative distribution function (CDF) plot showing the results of linear regression analysis of the distance of the 822 *RIM101*-dependent genes from a line of best fit for each strain comparison (Colored lines correspond to comparisons defined in legend). (F) Scatter plot displaying expression levels in Σ1278b(S2*RIM101*) relative to S288c wildtype (Red = *RIM101*-dependent genes). (G) Scatter plot displaying expression levels in Σ1278b *rim101Δ* relative to S288c wildtype (Red = *RIM101*-dependent genes).

We sought to distinguish whether the *RIM101* allele itself, or the *RIM101* pathway, imparts additional repressive capacity in S288c relative to ∑1278b by swapping *RIM101* alleles between backgrounds and assaying genome-wide expression by RNA-seq. Surprisingly, upon introduction of the non-native allele only three transcripts undergo statistically significant changes in expression in both backgrounds (Padj≤0.05) **(Figs 5A and S9)**. *AQY2*, *ncFRE6*, and *TIP1—*a cell surface mannoprotein—significantly change expression levels in response to incorporation of the non-native allele in both backgrounds. Such a focused, allele-dependent transcriptional response stands in stark contrast to other *trans*-regulators discovered in eQTL studies, which tend to affect expression of many genes. It remains unclear how only *AQY2*, *ncFRE6*, and *TIP1* are so dramatically influenced by interconversion of *RIM101* alleles between backgrounds. Moreover, the allele-dependent expression level of *TIP1* is surprising given that the *TIP1* allele and promoter region are invariant between S288c and ∑1278b, and especially because no change in *AQY2/ncFRE6* expression was observed in the ∑1278b *RIM101* deletion strain, nor was *TIP1* expression changed in the S288c *RIM101* deletion strain. (**S4 Table**). This high degree of allele-specificity suggests that unique combinations of factors can collaborate to regulate specific sets of genes.

Although only three transcripts become significantly differentially expressed in both S288c and ∑1278b *RIM101*-interconverted strains relative to their wildtype expression levels, many *RIM101*-dependent genes appear to be more highly expressed in S288c(∑*RIM101*) than in S288c wildtype, consistent with the S288c *RIM101* allele encoding a stronger repressor than the ∑1278b allele **(Fig 5A)**. In fact, in S288c(∑*RIM101*), expression levels of the 822 *RIM101*-dependent genes shift toward a pattern more similar to ∑1278b **(Fig 5B and 5C)**, partially phenocopying the expression shift observed in S288c *rim101∆* **(Fig 5B–5E).** However, incorporation of the strong S288c allele into ∑1278b does not result in a shift towards stronger repression of the same subset of genes **(Fig 5B, 5E, 5F and 5G)**. This asymmetry suggests that other background factors, and not solely the *RIM101* allele, are responsible for the gain of widespread *RIM101*-mediated repression in S288c, and that repression of *AQY2*, *ncFRE6* and *TIP1* is independent of such a background effect. These results imply that the same transcription factor can display drastically altered activity depending on the background that it is present within, and that certain backgrounds, such as ∑1278b, buffer against widespread transcriptional dysregulation upon introduction of a *RIM101* variant.

### A single nucleotide polymorphism within *RIM101* is necessary and sufficient for expression of both *AQY2* and *ncFRE6*

**Fig 6:**
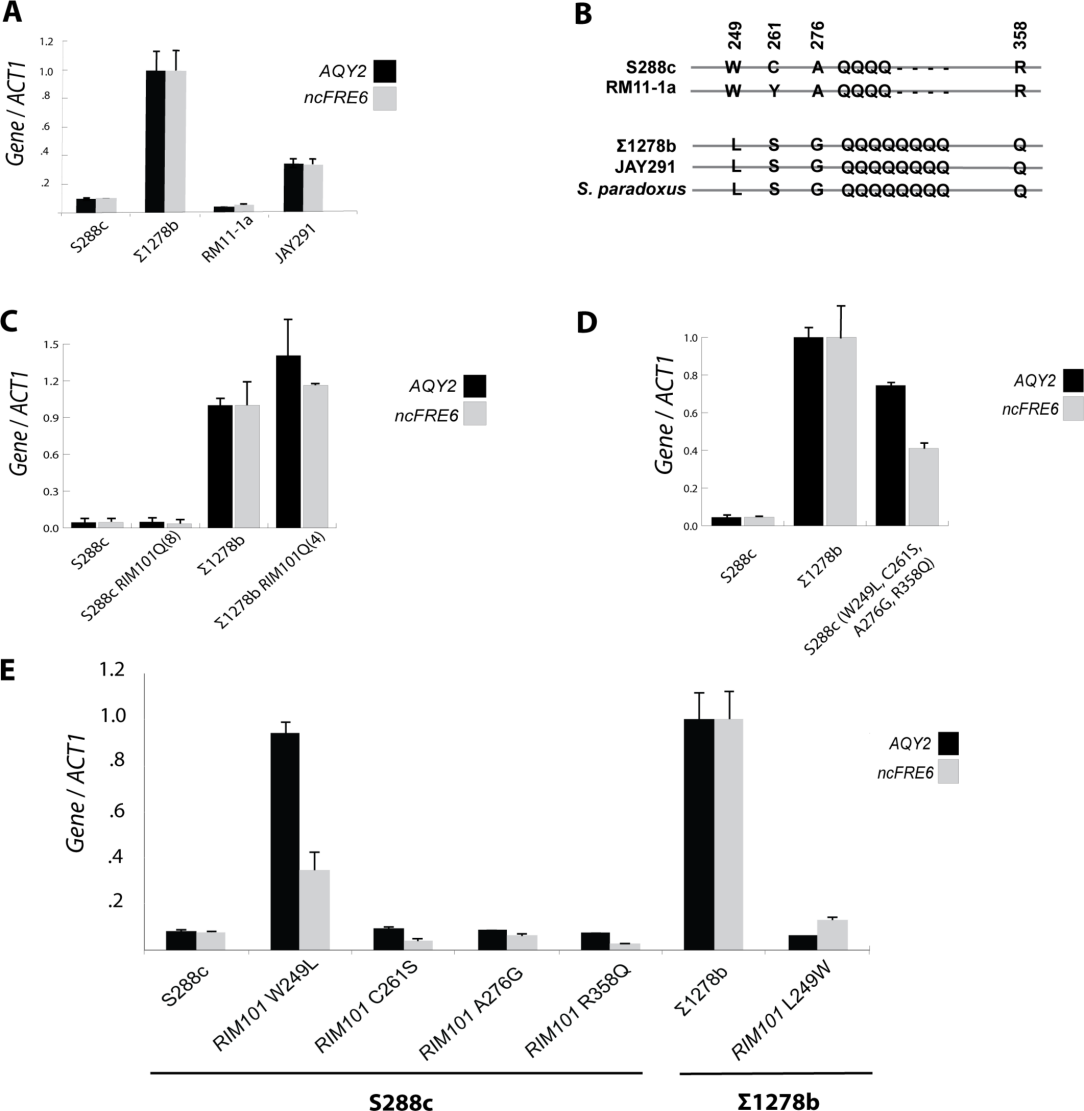
A single nucleotide polymorphism within *RIM101* is necessary and sufficient for expression of both *AQY2* and *ncFRE6*. (A) Relative expression of *AQY2* and *ncFRE6* in *S*. *cerevisiae* strains S288c, ∑1278b, RM11-1a, and JAY291 measured by qRT-PCR. (B) Sequence alignment of S288c, ∑1278b, RM11-1a, and JAY291 reveals features of *RIM101* conserved in *AQY2/ ncFRE6* expressing and non-expressing strains (Position numbers relative to S288c *Rim101* protein). (C) Relative expression of *AQY2* and *ncFRE6* in a polyQ-expanded S288c strain and a polyQ-truncated ∑1278b strain relative to each wt strain measured by qRT-PCR. (D) Relative expression of *AQY2* and *ncFRE6* in an S288c strain harboring four ∑1278b SNPs measured by qRT-PCR. (E) Relative expression of strains with individual *RIM101* point mutations measured by qRT-PCR.

Given that so few transcripts significantly change expression levels upon interconversion of *RIM101* alleles, we sought to better understand the molecular basis of such specificity. In order to infer the genomic feature or features within *RIM101* that contribute to repression of *AQY2* and *ncFRE6* in S288c, we screened two additional strains of *S*. *cerevisiae*, each harboring distinct combinations of sequence variation within *RIM101*, for expression of *AQY2* and *ncFRE6* **(Fig 6A)**. The *RIM101* DNA sequence includes 18 SNPs between S288c and Σ1278b, 13 of which alter the amino acid sequence of the *Rim101* protein **(S10 Fig)**. In addition to the 13 non-synonymous SNPs, a poly-glutamine repeat stretch is expanded from four amino acids in S288c to eight in Σ1278b. We selected RM11-1a [19] and JAY291 [44] for screening because they have distinct combinations of the sequence variations observed between S288c and Σ1278b. RM11-1a exhibits the *AQY2/ncFRE6*-repressed phenotype, suggesting that this strain harbors a *RIM101* allele capable of repressing the transcripts in a similar manner to S288c. However, the other strain, JAY291, expresses both transcripts as Σ1278b does. These results indicate that the two transcripts are expressed or repressed concurrently, implying the mechanism by which the transcripts are co-regulated is conserved across diverse *S*. *cerevisiae* strains.

We reasoned that the *RIM101* sequence necessary for repression must exist in both S288c and RM11-1a, but not in Σ1278b or JAY291. Alignment of the amino acid sequences of *Rim101* across the strains revealed four non-synonymous SNPs and a truncated poly-glutamine stretch that exist solely in the repressive strains **(Fig 6B)**. We sought to identify one or more of these variants between S288c and Σ1278b that control expression of *AQY2/ncFRE6*. Because variable length poly-glutamine tracks have been associated with altered protein structure and function, including altered protein-protein interactions [45], we first tested whether poly-glutamine repeat length affected *RIM101*-mediated repression. After expanding the poly-glutamine tract in S288c and truncating it in Σ1278b, we tested for expression of *AQY2/ncFRE6* and detected no deviation from either wildtype strain, suggesting that the length of the poly-glutamine tract does not, by itself, affect *RIM101* activity at this locus **(Fig 6C)**. Next we tested whether the four conserved amino acids are sufficient to affect *Rim101*-mediated repression of *AQY2/ncFRE6*. Indeed the collection of all four mutations is sufficient to rescue expression in S288c **(Fig 6D)**. Replacing each amino acid individually revealed one critical amino acid residue with regards to regulation of *AQY2/ncFRE6*. In S288c, W249L is sufficient to de-repress *AQY2* and *ncFRE6* **(Fig 6E)**. Furthermore, L249W is sufficient to repress *AQY2/ncFRE6* in Σ1278b. Hence, a single nucleotide polymorphism within the *RIM101* transcription factor determines whether *AQY2* and *ncFRE6* are expressed.

Finally, we sought to determine whether the amino acid present at position 249 is predictive of expression of *AQY2/ncFRE6* in other strains. We could predict expression of *AQY2/ncFRE6* in all five additional strains that we tested **(S11 Fig)**. Strains with L249 express *AQY2/ncFRE6*, and those with W249 do not. Clearly, position 249 within the *Rim101* protein is intimately linked to expression of *AQY2/ncFRE6* across a diverse array of strains. However, the ability to predict *AQY2/ncFRE6* expression is not conserved in a closely related species, *S*. *paradoxus* (**S12 Fig**). Hence, the effect of the W249L *RIM101* mutation appears to be clade specific, indicating that it may be a recently evolved regulatory mechanism.

## Discussion

We discovered a *trans*-regulatory single nucleotide polymorphism within the transcription factor *RIM101* that causes strain-specific expression of a pair of co-regulated, divergently oriented transcripts, *AQY2* and *ncFRE6*. Subsequent RNA-seq analysis of *RIM101* deletion strains revealed that *RIM101* controls expression of many more targets in S288c than ∑1278b, and suggests that the majority of differential expression between the two strains is related to differences in the *RIM101* pathway. Swapping *RIM101* alleles between S288c and ∑1278b strongly affects expression of only three transcripts in both strains: *AQY2*, *ncFRE6*, and *TIP1*. However, consistent with results from *RIM101* deletion strains, hundreds of other *RIM101*-dependent genes underwent subtle changes in expression specifically in the S288c background and not in ∑1278b.

### Dissection of a regulatory circuit uncovers principles contributing to the complexity of gene-expression regulation

Our study highlights the complexity of transcriptional regulation, even at a single locus. For example, though *Reb1* binding is clearly regulated by a *cis* variant between S288c and ∑1278b, and its binding pattern correlates with expression of *ncFRE6*, *Reb1* binding does not affect expression of *ncFRE6*. This result underscores the importance of single locus studies for identifying the true sources of differential expression, rather than relying on correlations between TF binding and expression. Furthermore, our results provide a unique example of how *cis*- and *trans*-linked DNA elements function in concert to affect gene expression. While both S288c and ∑1278b *cis* contexts are capable of directing expression of *AQY2/ncFRE6*, promoter activity is only apparent in the absence of an epistatic *trans*-factor that we determined to be the transcription factor *Rim101*.

Although our study initially focused on transcriptional regulation of a single locus in *cis*, much of the complexity governing the genome-wide regulatory capacity of *RIM101* arises from unknown background-dependent interactions that result in widespread differences in gene expression in *trans*. *RIM101* target genes undergo a widespread shift in expression pattern specifically in S288c(∑*RIM101*) but not in ∑1278b(S2*RIM101*). While the physical mechanism underlying this asymmetric response is unknown, previous *RIM101*-based research could offer clues. In particular, *Rim101* is extensively post-translationally modified, including by phosphorylation [46] and proteolytic processing [47]. It is not known whether a background-specific, allele-dependent *RIM101* interaction influences either of these modifications. Also, W249L resides in close proximity to the C2H2 zinc finger DNA-binding domain of *Rim101*, raising the possibility that variation at this position could impact DNA binding in S288c, but not ∑1278b, perhaps endowing *Rim101* with altered regulatory capacity in certain genetic contexts.

How do alternate *RIM101* alleles achieve such remarkable target specificity? Interconversion of the *RIM101* alleles between strain backgrounds strongly impacts only three transcripts, *AQY2*, *ncFRE6*, and *TIP1*, while other genes remain largely unaffected. How W249L, a mutation that has not been previously described, permits such specificity, remains unclear, though it is likely that such a phenomenon arises from allele-specific interactions with other genetic elements. However, the limited impact of the *RIM101* allele on expression of other genes implies that if this is the case, the interaction is specific to *AQY2/ncFRE6* and *TIP1*. Because the change in expression of *AQY2/ncFRE6* occurs in the opposite direction as *TIP1* (*AQY2/ncFRE6* higher in ∑*RIM101* strains, *TIP1* lower in ∑*RIM101* strains), it is possible that the mechanisms by which W249L elicits such a focused response are different between the two loci. Furthermore, expression of *AQY2/ncFRE6* or *TIP1* did not change in the ∑1278b *RIM101* deletion or the S288c *RIM101* deletion strains, respectively, further supporting a role for a W249L-specific interaction with other factors to influence *AQY2/ncFRE6* and*TIP1* expression specifically. Our results suggest that subtle mutations within TFs interact with genetic backgrounds to elicit unique combinations of gene expression patterns, likely expanding the phenotypic diversity observed within a population.

Such a focused, allele-dependent transcriptional response to a TF-linked variant stands in contrast to most known *trans*-regulators that affect expression of many genes [48]. In order to understand the mechanisms by which such subtle mutations affect target selection, it may be necessary to undertake a systematic allele-swapping strategy. Such studies are likely to reveal concepts important not only for understanding the biochemical nature of the variant itself, but also how the effect of the variant is propagated within alternate genetic backgrounds. Moreover, such an approach would afford researchers the ability to learn specifically about how variants within TFs, rather than other categories of genes typically discovered in eQTL studies [48], affect gene expression. Our finding that a SNP within a TF that regulates hundreds of genes causes large-scale expression differences in so few transcripts supports a model in which specific TF alleles interact in a combinatorial manner to regulate specific sets of genes [48].

One outstanding question is whether our findings regarding background- or allele-dependent activities of a transcription factor will be generalizable to other complex biological systems, including those involved in disease. For instance, transcription factors, including zinc finger TFs, are frequently mutated in cancers [49] and other human diseases, yet little is known about how the mutations relate to disease progression or outcome. With an enormous amount of sequence and functional data now available through consortiums such as The Cancer Genome Atlas (TCGA) and the 1,000 Genomes Project [50], tools now exist to test whether different alleles of the same TF can lead to variable expressivity of disease-associated phenotypes by impacting transcriptional profiles.

### Complex genetic interactions and evolution of the *RIM101* transcriptional regulatory network

*RIM101*-mediated regulation is affected not only by the *RIM101*-allele, but also the background that it is present within, suggesting that even in the relatively simple case of *RIM101*-mediated regulation of *AQY2/ncFRE6*, the regulatory pathways have diverged between S288c and ∑1278b. Furthermore, *S*. *paradoxus*, a species closely related to *S*. *cerevisiae*, does not conform to the same regulatory paradigm that controls *AQY2/ncFRE6* expression in the *S*. *cerevisiae* strains we tested. *AQY2/ncFRE6* expression is absent in *S*. *paradoxus* although it harbors a *RIM101* allele that includes the *S*. *cerevisiae* expression-permissive *Rim101* L249 variant. This suggests that the *RIM101*-dependent transcriptional regulatory circuit has been rewired at this locus. Clearly, the regulatory pathways underlying even simple, binary expression patterns display extraordinary complexity that could contribute to the plasticity of gene expression regulation observed throughout evolution.

The *RIM101* allele-dependent interactions that we observed may contribute to the phenotypic diversity observed between S288c and ∑1278b. Because *AQY2* is non-functional in S288c, but functional in ∑1278b, the evolutionary pressures affecting expression of *AQY2* are likely different between the strains. Perhaps the subtle *RIM101* W249L variant, which strongly alters expression of only three transcripts, represents an example of genetic drift between the strains.*TIP1* is a cell-surface mannoprotein and *AQY2* is a cell surface water channel, raising the possibility that the focused *AQY2* and *TIP1* expression differences caused by W249L may result in an altered cell surface environment between the strains. Although we showed that the *RIM101* allele did not affect haploid invasive growth, such a re-structuring of the cell surface could result in other *RIM101*, *AQY2*, or *TIP1*-linked cell surface phenotypes.

Cryptic genetic variation (CGV) is genetic variation that influences a phenotype in certain environmental or genetic contexts, but not in others [51]. Although it is almost certain that CGV is common in nature, very few examples have been described in detail [52, 53]. Our study highlights a previously undescribed mechanism by which CGV can manifest. We propose that polymorphic transcription factors likely represent a source of CGV whereby certain genetic backgrounds buffer against widespread transcriptional dysregulation upon introduction of a non-native allele (as in ∑1278b(S2*RIM101*)), while others are subject to a dramatic shift in gene expression (as in S288c(∑*RIM101*). The regulatory capacity of *RIM101* is highly background-dependent and the interaction of *RIM101* with genetic background determines whether a cell will undergo widespread or localized changes in its transcriptional program upon introduction of an alternative *RIM101* allele.

## Materials and Methods

### Strains, media, mirobiological techniques, and growth conditions

*S*. *cerevisiae* strains used in this study were derived from BY4742 (S288c, his3∆1, lys2∆0, leu2∆0, ura3∆0) or L6441 (Σ1278b, ura3-52, leu2::hisG, his3::hisG). Other strains used in Figs 6A and S11 including JAY291, RM11-1a, CLIB324, CLIB382, YPS163, T7, and UC5 are homothallic diploids generously donated by Justin Fay (Washington University). The *delitto perfetto* [36] method was used to edit genome sequences. For gene expression experiments, cells were grown in standard YPD media to mid-log phase in YPD before RNA isolation. Primers and plasmids used in this study are listed in supplementary materials and methods. Invasive growth phenotype assay (S7 Fig) was performed as described in [43] by patching cells onto a YPD plate for two days and washing the plate under gently running water before imaging.

### qRT-PCR

RNA was extracted using a standard acid phenol chloroform extraction (Collart, 2001) and DNased with RQ1 DNase (Promega #M6106) according to manufacturer’s instructions. 1ug of RNA was reverse transcribed using Multiscribe reverse transcriptase (Thermo Fisher #4311235) with random hexamers, except for *ncFRE6*, for which we used a gene specific RT primer due to the need to measure RNA levels strand-specifically. cDNA was measured using targeted qPCR primers (S1 Text) and SYBR select (Life Technologies #4472908) on the Biorad CFX qPCR system.

### Genome-wide expression profiling by RNA-seq

Strand specific RNA-seq libraries were made using the NEBNext Ultra Strand-specific RNA-seq library prep kit (NEB #E7420S/L) with manufacturers instructions. Briefly, RNA was isolated by standard acid phenol chloroform extraction and poly-adenylated RNA was purified with oligo (dT) dynabeads (Thermo Fisher #61002). RNA was fragmented and first strand synthesis performed with ProtoScript II reverse transcriptase and random hexamers. Second strand synthesis then incorporated Uridine residues into cDNA. cDNA was purified with AMPure beads (Agencourt). cDNA was then dA-tailed and NEBNext adaptors for Illumina were ligated before another AMPure purification. USER excision removed the second strand and libraries were amplified with NEBNext High Fidelity PCR master mix (NEB). NEBNext Multiplex oligos 1–12 (NEB #E7335) were incorporated during PCR. Libraries were quantified with the Qubit (Life technologies) before pooling at equimolar concentrations and sequencing on an Illumina Hiseq. Reads were mapped using bowtie2 and differential expression was assessed using DEseq (S1 Text).

### Expression-guided bulked segregant analysis

S288c (BY4741) and Σ1278b (L6441) haploid strains were crossed to generate a heterozygous diploid. The diploid was sporulated on traditional sporulation media and tetrads were dissected. 28 haploid segregants of an S288c x Σ1278b cross were grown to mid-log phase in YPD and tested for expression of *AQY2/ncFRE6* by qRT-PCR. Segregants were binned based on whether they expressed *AQY2/ncFRE6* or not. Genomic DNA was isolated, treated with RNAse A (Thermo Fisher #AM2270), and purified by phenol chloroform extraction and ethanol precipitation. DNA concentrations were measured with the Qubit (Thermo Fisher #Q33216) and pooled at 10nM separately for strains either expressing or not expressing *AQY2/ncFRE6*. DNA was sheared using the Covaris M220 ultrasonicator to an average size of 500 bp. DNA was blunted and dA-tailed before ligation of Illumina sequencing adapters. Libraries were amplified by Phusion polymerase with Illumina multiplex barcodes 1+2 for ten cycles before analysis on the Bioanalyzer (Agilent). Samples were sequenced on an Illumina HiSeq 2000. Reads were mapped using bowtie2 and variants identified using GATK (S1 Text).

## Supporting Information

S1 Fig Most transcripts are expressed at similar levels between S288c and ∑1278b. (A) Integrated Genome Viewer (IGV) (https://www.broadinstitute.org/igv/) screenshot showing a representative region (100kb) of the genome between S288c (Red) and ∑1278b (Blue). Data are displayed as positive (Watson) strand above the axis and negative (Crick) strand below the axis. Black boxes = differentially expressed transcripts between S288c and ∑1278b. (B) Scatter plot displaying expression levels of 5682 genes in S288c relative to ∑1278b. Red dots (n = 1207) are significantly differentially expressed between the wildtype strains (DESeq Padj 0.0005). (C) Scatter plot of antisense transcripts for 5682 genes between S288c and ∑1278b.
(TIF)

S2 Fig *Reb1* binding is controlled by a SNP but does not cause expression of *ncFRE6*. (A) Venn diagram displaying conservation of *Reb1* binding events between S288c and ∑1278b (Red = number of S288c-unique *Reb1* binding events, Green = number of conserved *Reb1* binding events, Blue = number of ∑1278b-unique *Reb1* binding events). (B) *Reb1* binding motifs derived from MEME for S288c and ∑1278b (Red box = *ncFRE6*-associated variable allele between S288c (A) and ∑1278b (T)). (C) IGV screenshot displaying strand–specific RNA-seq of the *ncFRE6* region in S288c (Red) and ∑1278b (Blue). Data are displayed as positive (Watson) strand above the axis and negative (Crick) strand below the axis. *Reb1* ChIP-seq data in black for S288c and ∑1278b. Location of a single nucleotide polymorphism within a canonical *Reb1* binding site is represented by a red line. (D) ChIP-qPCR displaying relative *Reb1* occupancy at the location of a *Reb1* binding site in S288c, ∑1278b and SNP-interconverted strains. Data normalized to input for each strain. (E) qRT-PCR showing levels of *ncFRE6* in S288c, ∑1278b and *Reb1* SNP-interconverted strains.
(TIF)

S3 Fig The *AQY2/ncFRE6* promoter is highly variable between S288c and ∑1278b. Histogram comparing the number of promoters (Y axis), ranked by SNP density (X axis).
(TIF)

S4 Fig Schematic showing the workflow for expression-guided bulked segregant analysis (eBSA). Briefly, segregants were binned based on whether they express *AQY2/ncFRE6*. Genomic DNA from the expressing group was pooled separately from genomic DNA from the non-expressing pool. Pools were sequenced and reads mapped to both the S288c and ∑1278b genomes. The allelic frequency for each single nucleotide polymorphism is quantified based on read counts mapping to each genome for each pool. A region where only S288c alleles exist in one pool (i.e. expressors) and only ∑1278b alleles exist in the other (i.e. non-expressors) harbor the variant driving differential expression of the transcript being interrogated (i.e. *AQY2/ncFRE6*).
(TIF)

S5 Fig Expression-guided bulked segregant analysis maps the *trans* factor to the left arm of chromosome VIII. Scatter plots displaying the allelic frequencies of every SNP between S288c and ∑1278b within pools of genomic DNA from either expressing or non-expressing segregants (Red = expressing, Blue = non-expressing). Plots are arranged by chromosome and genome mapped against. X-axis is position along the chromosome. Y-axis is allelic frequency. Red dots represent SNP frequency within the expressing pools.
(TIF)

S6 Fig *Rim101* is one of the most sequence-variable transcription factors between S288c and ∑1278b. Histogram displays the number of single nucleotide polymorphisms in 249 DNA-binding proteins. X-axis represents number of non-synonomous SNPs/kb. Y-axis is number of transcription factors.
(TIF)

S7 Fig The S288c *RIM101* allele complements the ∑1278b *RIM101* allele in ∑1278b for the invasive growth phenotype. Strains were patched to YPD for two days and washed with gently running water before imaging.
(TIF)

S8 Fig Deletion of *RIM101* results in an asymmetric transcriptional response between S288c and ∑1278b deletion and wildtype strains. CDF plot examining the impact of deleting *RIM101* on 822 *RIM101* targets in (A) S288c or (B) ∑1278b. Y-axis represents percentage of *RIM101* targets. X-axis represents cumulative differential expression for each comparison. Comparisons specified using S2 (S288c) and ∑ (∑1278b) as abbreviations.
(TIF)

S9 Fig *ncFRE6* expression is controlled by the *RIM101* allele and is unaffected by deletion of *RIM101* in ∑1278b. (A) Scatter plot displaying expression levels for each antisense transcript (dots) in S2(*∑RIM101*) relative to S288c wildtype (Red = *RIM101*-dependent genes as defined in Fig 4). (B) Scatter plot displaying expression levels for each antisense transcript in ∑1278b(S2*RIM101*) relative to ∑1278b wildtype. (C) Scatter plot displaying expression levels for each antisense transcript in S288c *rim101∆* relative to S288c wildtype. (D) Scatter plot displaying expression levels for each antisense transcript in ∑1278b *rim101∆* relative to ∑1278b wildtype.
(TIF)

S10 Fig *Rim101* protein sequence is highly variable between S288c and ∑1278b. ClustalW protein alignment of *Rim101* showing 13 amino acid substitutions and a truncated poly-glutamine tract in S288c relative to ∑1278b.
(TIF)

S11 Fig Position 249 within the *Rim101* protein determines the on/off state of *AQY2/ncFRE6* in five additional strains of *S*. *cerevisiae*, but not in *S*. *paradoxus*. (A) qRT-PCR of *AQY2* (Black) and *ncFRE6* (grey) in five additional strains of *S*. *cerevisiae*. (B) Alignment of the region of *Rim101* implicated in repression of *AQY2/ncFRE6*. (C) qRT-PCR of *AQY2* and *ncFRE6* in S288c, ∑1278b, and *S*. *paradoxus*.
(TIF)

S1 Table Differential expression summary for all pairwise comparisons, overlaps between select comparisons, and on/off transcript assessment at varying cutoffs. (XLSX)

S2 Table GO terms (molecular function) for S288c vs ∑1278b significantly differentially expressed genes (pval ≤ 0.002). (XLSM)

S3 Table List of potential TF binding sites disrupted by SNPs in S288c or ∑1278b within and surrounding the *AQY2/ncFRE6* intergenic region. (XLSX)

S4 Table Complete differential expression results for 5682 protein-coding genes after removal of dubious ORFs and genes not present in both strains. Tabs are arranged by comparison. Includes all interstrain comparisons: 1) S288c wt vs. ∑1278b wt, 2) S288c *rim101∆* vs. ∑1278b *rim101∆*, 3) S288c(∑*RIM101*) vs. ∑1278b(S2*RIM101*). Also includes all intrastrain comparisons: 4) S288c wt vs. S288c *rim101∆*, 5) ∑1278b wt vs. ∑1278b *rim101∆*, 6) S288c wt vs. S288c(∑*RIM101*), and 7) ∑1278b wt vs. ∑1278b(S2 *RIM101*).
(XLSX)

S5 Table Full differential expression results for 5682 antisense transcripts after removal of dubious ORFs and genes not present in both strains. Tabs are arranged by comparison. Includes all interstrain comparisons: 1) S288c wt vs. ∑1278b wt, 2) S288c *rim101∆* vs. ∑1278b *rim101∆*, 3) S288c(∑ *RIM101*) vs. ∑1278b(S2 *RIM101*). Also includes all intrastrain comparisons: 4) S288c wt vs. S288c *rim101∆*, 5) ∑1278b wt vs. ∑1278b *rim101∆*, 6) S288c wt vs. S288c(∑*RIM101*), and 7) ∑1278b wt vs. ∑1278b(S2 *RIM101*).
(XLSX)

S1 Text Supplemental experimental procedures. Extended description of procedures and sequencing statistics.
(DOCX)

## References

[1] Stranger BE, Stahl E a., Raj T. Progress and promise of genome-wide association studies for human complex trait genetics. Genetics. 2011;187(2):367–83. 10.1534/genetics.110.120907 21115973 PMC3030483

[2] Welter D, MacArthur J, Morales J, Burdett T, Hall P, Junkins H, et al The NHGRI GWAS Catalog, a curated resource of SNP-trait associations. Nucleic Acids Res. 2014;42(D1):1001–6. 10.1093/nar/gkt1229 PMC3965119 24316577

[3] Ramos EM, Din-Lovinescu C, Berg JS, Brooks LD, Duncanson A, Dunn M, et al Characterizing genetic variants for clinical action. Am J Med Genet Part C Semin Med Genet. 2014;166(1):93–104. 10.1002/ajmg.c.31386 PMC4158437 24634402

[4] Maurano MT, Humbert R, Rynes E, Thurman RE, Haugen E, Wang H, et al Systematic Localization of Common Disease-Associate Variation in Regulatorty DNA. Science (80). 2012;337(September):1190. 10.1126/science.1222794 PMC3771521 22955828

[5] Hindorff L a, Sethupathy P, Junkins H a, Ramos EM, Mehta JP, Collins FS, et al Potential etiologic and functional implications of genome-wide association loci for human diseases and traits. Proc Natl Acad Sci U S A. 2009;106(23):9362–7. 10.1073/pnas.0903103106 19474294 PMC2687147

[6] Chandler CH, Chari S, Dworkin I. Does your gene need a background check? How genetic background impacts the analysis of mutations, genes, and evolution. Trends Genet. 2013; Jun;29(6):358–66. 10.1016/j.tig.2013.01.009 23453263 PMC3692003

[7] Chandler CH. Cryptic intraspecific variation in sex determination in Caenorhabditis elegans revealed by mutations. Heredity. 2010;105(5):473–82. 10.1038/hdy.2010.62 20502478

[8] Matin A, Nadeau JH. Sensitized polygenic trait analysis. Trends Genet. 2001;17(12):727–31. 11718927 10.1016/s0168-9525(01)02528-8

[9] Nadimpalli S, Persikov A V., Singh M. Pervasive Variation of Transcription Factor Orthologs Contributes to Regulatory Network Evolution. PLOS Genet. 2015;11(3) 10.1371/journal.pgen.1005011 PMC4351887 25748510

[10] Kim J, He X, Sinha S. Evolution of regulatory sequences in 12 Drosophila species. PLoS Genet. 2009;5(1). 10.1371/journal.pgen.1000330 PMC2607023 19132088

[11] Cubillos FA, Billi E. Assessing the complex architecture of polygenic traits in diverged yeast populations. Molecular Ecology. 2011;1401–13. 10.1111/j.1365-294X.2011.05005.x 21261765

[12] Perou CM, Sørlie T, Eisen MB, van de Rijn M, Jeffrey SS, Rees C a, et al Molecular portraits of human breast tumours. Nature. 2000;406(May):747–52. 10963602 10.1038/35021093

[13] Golub TR, Slonim DK, Tamayo P, Huard C, Gaasenbeek M, Mesirov JP, et al Molecular classification of cancer: class discovery and class prediction by gene expression monitoring. Science. 1999;286(5439):531–7. 10521349 10.1126/science.286.5439.531

[14] Litvin O, Schwartz S, Wan Z, Schild T, Rocco M, Oh NL, et al Interferon α/β Enhances the Cytotoxic Response of MEK Inhibition in Melanoma. Mol Cell. 2015;57:784–96. 10.1016/j.molcel.2014.12.030 25684207 PMC4355234

[15] Calon A, Lonardo E, Berenguer-llergo A, Espinet E, Hernando-momblona X, Iglesias M, et al Stromal gene expression defines poor-prognosis subtypes in colorectal cancer. Nat Genet 2015;47(February):320–9. 10.1038/ng.3225 25706628

[16] Dworkin I, Kennerly E, Tack D, Hutchinson J, Brown J, Mahaffey J, et al Genomic consequences of background effects on scalloped mutant expressivity in the wing of drosophila melamogaster. Genetics. 2009;181(3):1065–76. 10.1534/genetics.108.096453 19064709 PMC2651043

[17] Gallagher JEG, Zheng W, Rong X, Miranda N, Lin Z, Dunn B, et al Divergence in a master variator generates distinct phenotypes and transcriptional responses. Genes and Dev. 2014;409–21. 10.1101/gad.228940.113 24532717 PMC3937518

[18] Schadt EE, Monks SA, Drake TA, Lusis AJ, Che N, Colinayo V, et al Genetics of gene expression surveyed in maize, mouse and man. Nature. 2003;205(October 2002):1–6. 10.1038/nature01434 12646919

[19] Brem RB, Clinton R. Genetic Dissection of Transcriptional Regulation in Budding Yeast. Science. 2002;296(April):752–6. 11923494 10.1126/science.1069516

[20] Jansen RC, Nap JP. Genetical genomics: The added value from segregation. Trends Genet. 2001;17(7):388–91. 11418218 10.1016/s0168-9525(01)02310-1

[21] Nica A, Dermitzakis E. Expression quantitative trait loci: present and future. Philos Trans R Soc B. 2013;1(May). 10.1098/rstb.2012.0362 PMC3682727 23650636

[22] Rockman M V, Kruglyak L. Genetics of global gene expression. Nat Rev Genet. 2006;11;7(11):862–72. 17047685 10.1038/nrg1964

[23] Westra H-J, Peters MJ, Esko T, Yaghootkar H, Schurmann C, Kettunen J, et al Systematic identification of trans eQTLs as putative drivers of known disease associations. Nat Genet. 2013;45(10):1238–43. 10.1038/ng.2756 24013639 PMC3991562

[24] Westra H-J, Franke L. From genome to function by studying eQTLs. Biochim Biophys Acta. 2014;1842(10):1896–902. 10.1016/j.bbadis.2014.04.024 24798236

[25] Stranger BE, Nica AC, Forrest MS, Dimas A, Bird CP, Beazley C, et al Population genomics of human gene expression. Nat Genet. 2007;39(10):1217–24. 17873874 10.1038/ng2142 PMC2683249

[26] Göring HHH, Curran JE, Johnson MP, Dyer TD, Charlesworth J, Cole S a, et al Discovery of expression QTLs using large-scale transcriptional profiling in human lymphocytes. Nat Genet. 2007;39(10):1208–16. 17873875 10.1038/ng2119

[27] Montgomery SB, Dermitzakis ET. From expression QTLs to personalized transcriptomics. Nat Rev Genet. 2011;12(4):277–82. 10.1038/nrg2969 21386863

[28] Pickrell JK. Joint analysis of functional genomic data and genome-wide association studies of 18 human traits. Am J Hum Genet. 2014;94(4):559–73. 10.1016/j.ajhg.2014.03.004 24702953 PMC3980523

[29] Battle A, Mostafavi S, Zhu X, Potash JB, Weissman MM, McCormick C, et al Characterizing the genetic basis of transcriptome diversity through RNA-sequencing of 922 individuals. Genome Res. 2014;24(1):14–24. 10.1101/gr.155192.113 24092820 PMC3875855

[30] Rudolph H, Hinnen a. The yeast PHO5 promoter: phosphate-control elements and sequences mediating mRNA start-site selection. Proc Natl Acad Sci U S A. 1987;84(5):1340–4. 2881299 10.1073/pnas.84.5.1340 PMC304424

[31] Houseley J, Rubbi L, Grunstein M, Tollervey D, Vogelauer M. A ncRNA modulates histone modification and mRNA induction in the yeast GAL gene cluster. Mol Cell. 2008;32(5):685–95. 10.1016/j.molcel.2008.09.027 19061643 PMC7610895

[32] Hainer SJ, Pruneski J a, Mitchell RD, Monteverde RM, Martens J a. Intergenic transcription causes repression by directing nucleosome assembly. Genes Dev. 2011;25(1):29–40. 10.1101/gad.1975011 21156811 PMC3012934

[33] Hongay CF, Grisafi PL, Galitski T, Fink GR. Antisense Transcription Controls Cell Fate in Saccharomyces cerevisiae. Cell. 2006;127(4):735–45. 17110333 10.1016/j.cell.2006.09.038

[34] Xu Z, Wei W, Gagneur J, Clauder-Münster S, Smolik M, Huber W, et al Antisense expression increases gene expression variability and locus interdependency. Mol Syst Biol. 2011;7(468):468. 21326235 10.1038/msb.2011.1 PMC3063692

[35] Dowell RD, Ryan O, Jansen A, Cheung D, Agarwala S, Danford T, et al Genotype to Phenotype : A Complex Problem. Science. 2010; 328 (5977):469 10.1126/science.1189015 20413493 PMC4412269

[36] Storici F, Lewis LK, Resnick MA. In vivo site-directed mutagenesis using oligonucleotides. Nat. Biotechnol. 2001;19:773–6. 11479573 10.1038/90837

[37] Carbrey JM, Bonhivers M, Boeke JD, Agre P. Aquaporins in Saccharomyces: Characterization of a second functional water channel protein. Proc Natl Acad Sci U S A. 2001;1 30;98(3):1000–5. 11158584 10.1073/pnas.98.3.1000 PMC14698

[38] Winge O, Laustsen O. On two types of spore germination, and on genetic segregations in Saccharomyces demonstrated through single spore cultures. Cr Trav Lab Carlsb Ser Physiol. 1937;(24):263–315.

[39] Kesseli R V. Identification of markers linked to disease-resistance genes by bulked segregant analysis : A rapid method to detect markers in specific genomic regions by using segregating populations. Proc. Natl. Acad. Sci. USA. 1991;88(November):9828–32. 1682921 10.1073/pnas.88.21.9828 PMC52814

[40] Ehrenreich IM, Torabi N, Jia Y, Kent J, Martis S, Shapiro J a, et al Dissection of genetically complex traits with extremely large pools of yeast segregants. Nature. 2010;464(7291):1039–42. 10.1038/nature08923 20393561 PMC2862354

[41] Winzeler E a, Shoemaker DD, Astromoff a, Liang H, Anderson K, Andre B, et al Functional characterization of the S. cerevisiae genome by gene deletion and parallel analysis. Science. 1999;285(5429):901–6. 10436161 10.1126/science.285.5429.901

[42] Ryan O, Shapiro RS, Kurat CF, Mayhew D, Baryshnikova a., Chin B, et al Global Gene Deletion Analysis Exploring Yeast Filamentous Growth. Science. 2012;337(6100):1353–6. 10.1126/science.1224339 22984072

[43] Gimeno CJ, Ljungdahl PO, Styles C a., Fink GR. Unipolar cell divisions in the yeast S. cerevisiae lead to filamentous growth: Regulation by starvation and RAS. Cell. 1992;68(6):1077–90. 1547504 10.1016/0092-8674(92)90079-r

[44] Argueso JJL, Carazzolle MMF, Mieczkowski P a, Duarte FM, Netto OVC, Missawa SK, et al Genome structure of a Saccharomyces cerevisiae strain widely used in bioethanol production. Genome Res. 2009;19:2258–70. 10.1101/gr.091777.109 19812109 PMC2792172

[45] Schaefer MH, Wanker EE, Andrade-Navarro M a. Evolution and function of CAG/polyglutamine repeats in protein-protein interaction networks. Nucleic Acids Res. 2012;40(10):4273–87. 10.1093/nar/gks011 22287626 PMC3378862

[46] Nishizawa M, Tanigawa M, Hayashi M, Maeda T, Yazaki Y, Saeki Y, et al Pho85 kinase, a cyclin-dependent kinase, regulates nuclear accumulation of the Rim101 transcription factor in the stress response of Saccharomyces cerevisiae. Eukaryot Cell. 2010;9(6):943–51. 10.1128/EC.00247-09 20382759 PMC2901641

[47] Weishi L, Mitchell AP. Proteolytic Activation of Rimlp, a Positive Regulator of Yeast Sporulation and Invasive Growth. Genetics. 1997;145:63–73. 9017390 10.1093/genetics/145.1.63 PMC1207785

[48] Yvert G, Brem RB, Whittle J, Akey JM, Foss E, Smith EN, et al Trans-acting regulatory variation in Saccharomyces cerevisiae and the role of transcription factors. Nature genetics. 2003;35(1): 57–64. 12897782 10.1038/ng1222

[49] Ashworth J, Bernard B, Reynolds S, Plaisier CL, Shmulevich I, Baliga NS. Structure-based predictions broadly link transcription factor mutations to gene expression changes in cancers. Nucleic Acids Research. 2014;42(21):12973–83. 10.1093/nar/gku1031 25378323 PMC4245936

[50] McVean G a., Altshuler (Co-Chair) DM, Durbin (Co-Chair) RM, Abecasis GR, Bentley DR, Chakravarti A, et al An integrated map of genetic variation from 1,092 human genomes. Nature. 2012;491(7422):56–65. 10.1038/nature11632 23128226 PMC3498066

[51] Paaby AB, Rockman M V. Cryptic genetic variation: evolution’s hidden substrate. Nat Rev Genet; 2014;15(4):247–58. 10.1038/nrg3688 24614309 PMC4737706

[52] Rutherford SL, Lindquist S. Hsp90 as a capacitor for morphological evolution. Nature. 1998;396(6709):336–42. 9845070 10.1038/24550

[53] Milloz J, Duveau F, Nuez I, Félix MA. Intraspecific evolution of the intercellular signaling network underlying a robust developmental system. Genes Dev. 2008;22(21):3064–75. 10.1101/gad.495308 18981482 PMC2577794

