# Supplementary figures and images for: A trans-acting Variant within the Transcription Factor RIM101 Interacts with Genetic Background to Determine its Regulatory Capacity
Source: PLoS Genet. 2016 Jan 11;12(1):e1005746. doi: 10.1371/journal.pgen.1005746 (PMC4709078; doi:10.1371/journal.pgen.1005746)

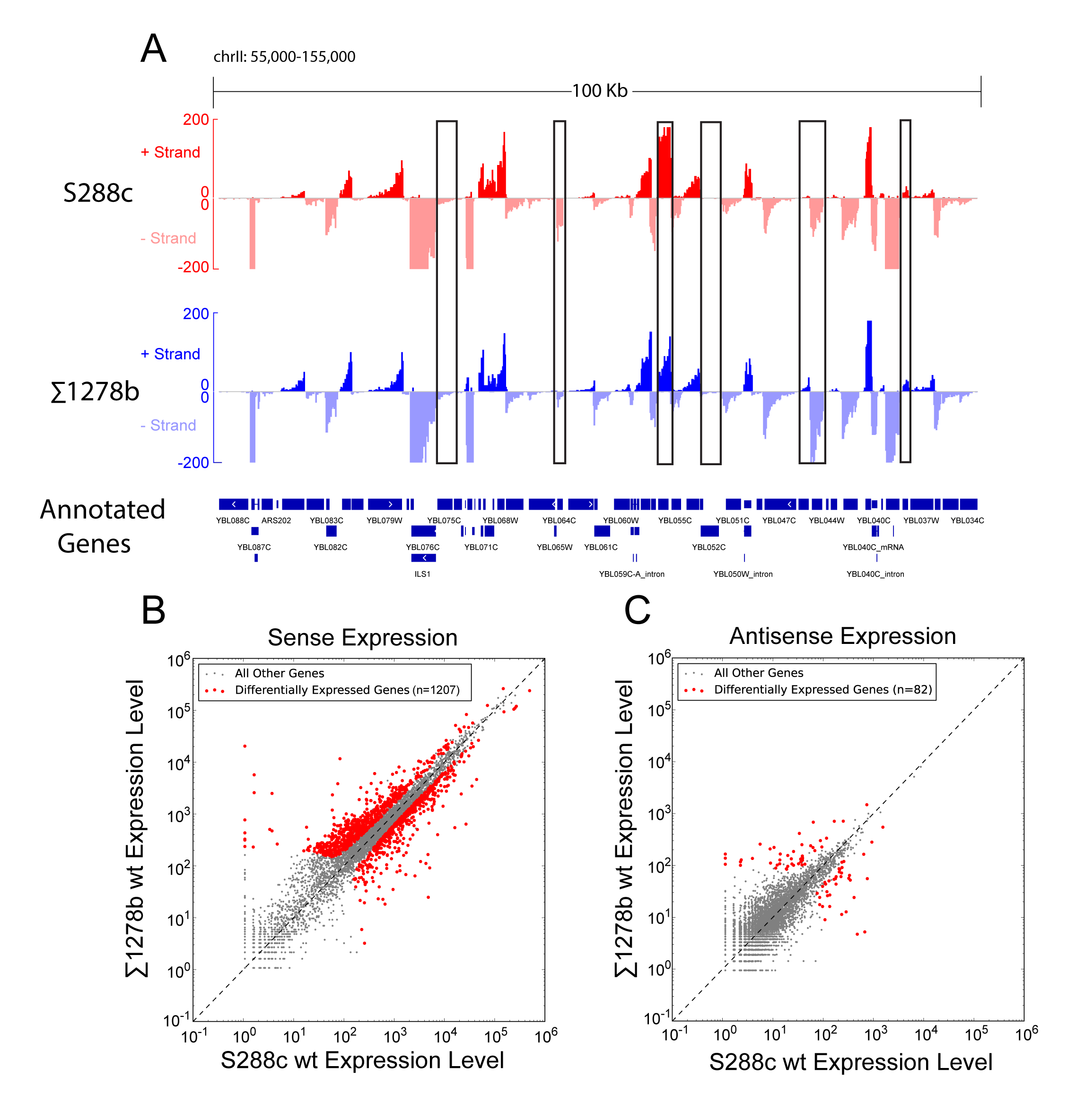

Supplement: S1 Fig — (A) Integrated Genome Viewer (IGV) (https://www.broadinstitute.org/igv/) screenshot showing a representative region (100kb) of the genome between S288c (Red) and ∑1278b (Blue). Data are displayed as positive (Watson) strand above the axis and negative (Crick) strand below the axis. Black boxes = differentially expressed transcripts between S288c and ∑1278b. (B) Scatter plot displaying expression levels of 5682 genes in S288c relative to ∑1278b. Red dots (n = 1207) are significantly differentially expressed between the wildtype strains (DESeq Padj 0.0005). (C) Scatter plot of antisense transcripts for 5682 genes between S288c and ∑1278b. (TIF) [file pgen.1005746.s001.tif]

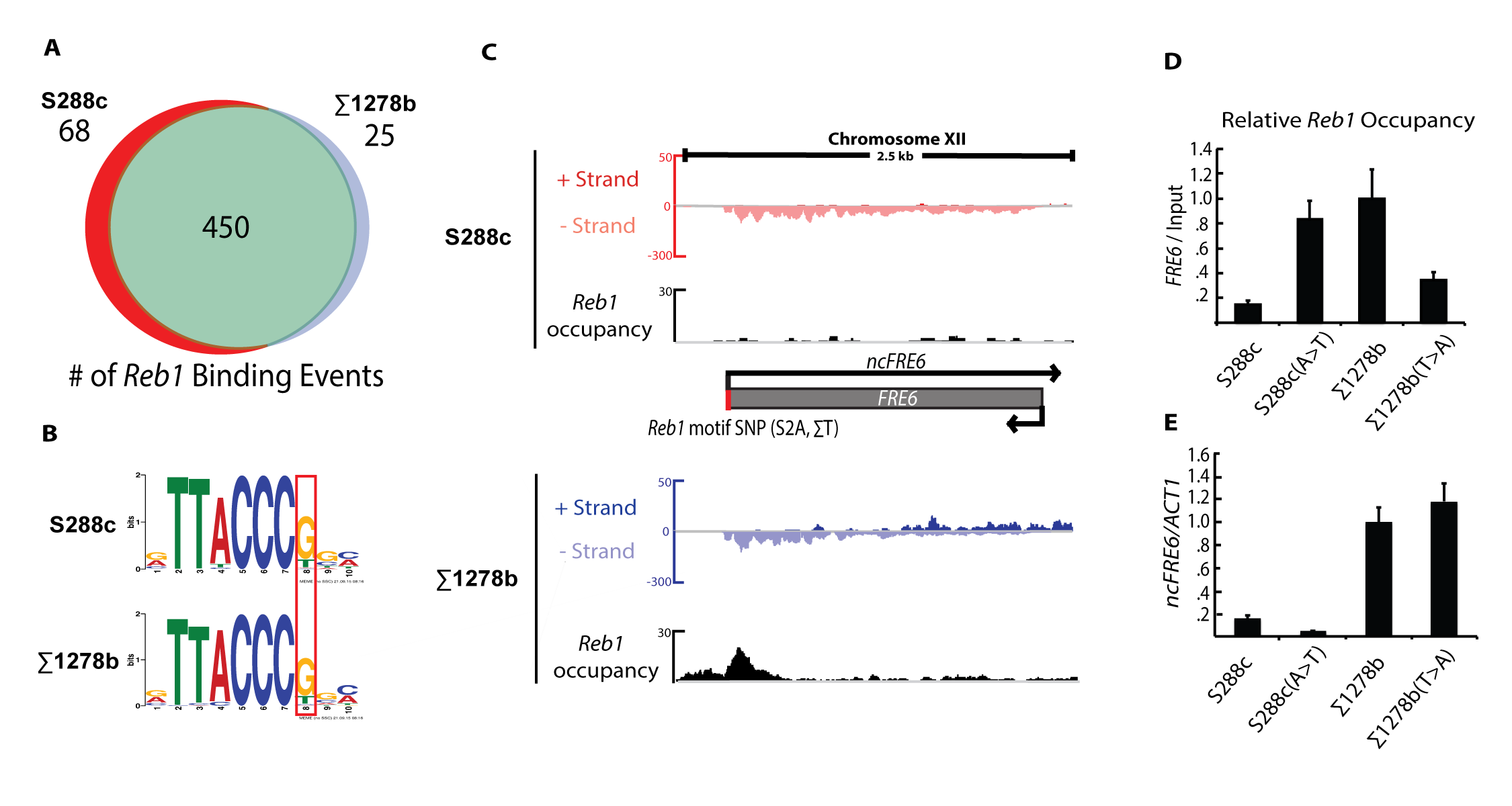

Supplement: S2 Fig — (A) Venn diagram displaying conservation of Reb1 binding events between S288c and ∑1278b (Red = number of S288c-unique Reb1 binding events, Green = number of conserved Reb1 binding events, Blue = number of ∑1278b-unique Reb1 binding events). (B) Reb1 binding motifs derived from MEME for S288c and ∑1278b (Red box = ncFRE6-associated variable allele between S288c (A) and ∑1278b (T)). (C) IGV screenshot displaying strand–specific RNA-seq of the ncFRE6 region in S288c (Red) and ∑1278b (Blue). Data are displayed as positive (Watson) strand above the axis and negative (Crick) strand below the axis. Reb1 ChIP-seq data in black for S288c and ∑1278b. Location of a single nucleotide polymorphism within a canonical Reb1 binding site is represented by a red line. (D) ChIP-qPCR displaying relative Reb1 occupancy at the location of a Reb1 binding site in S288c, ∑1278b and SNP-interconverted strains. Data normalized to input for each strain. (E) qRT-PCR showing levels of ncFRE6 in S288c, ∑1278b and Reb1 SNP-interconverted strains. (TIF) [file pgen.1005746.s002.tif]

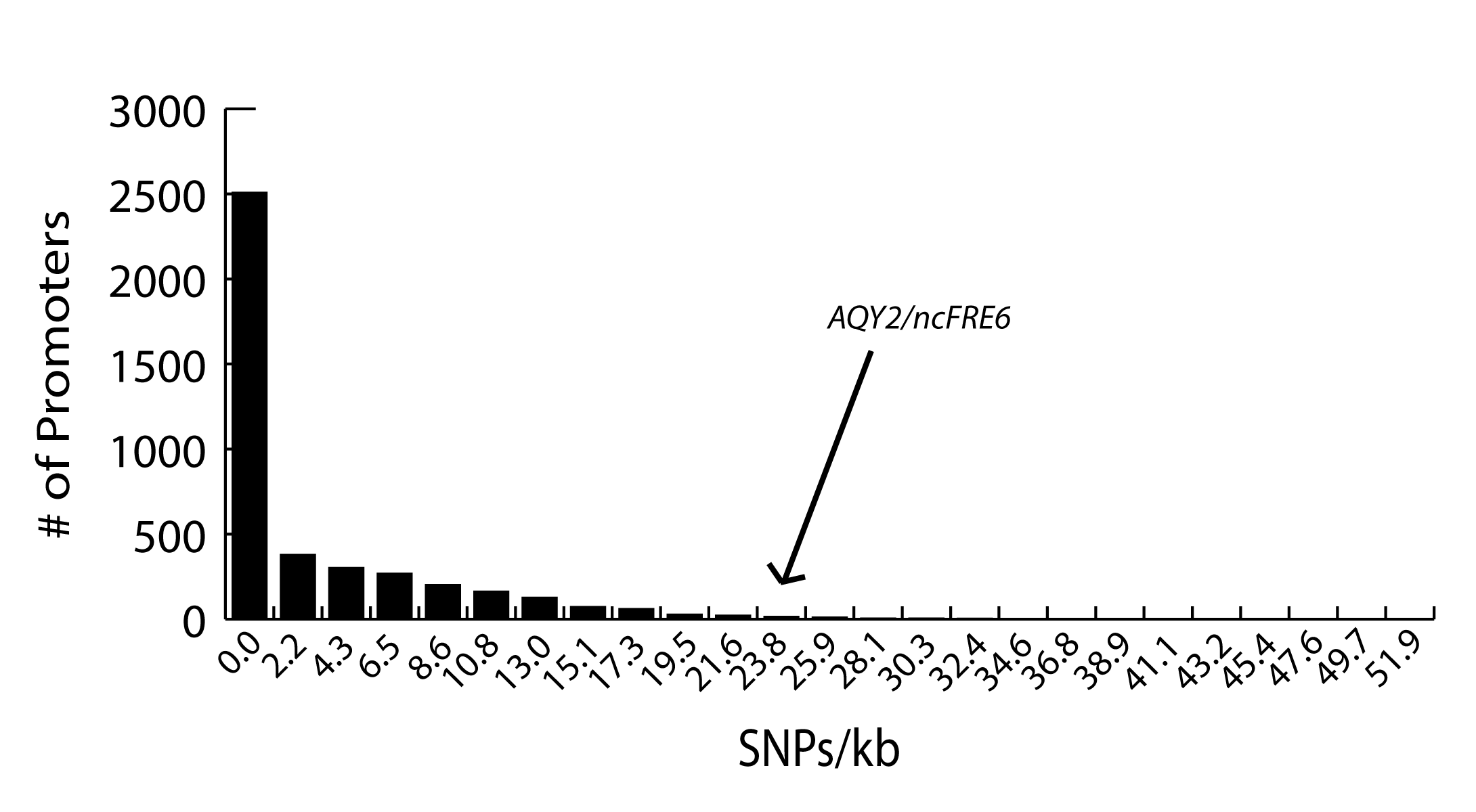

Supplement: S3 Fig — Histogram comparing the number of promoters (Y axis), ranked by SNP density (X axis). (TIF) [file pgen.1005746.s003.tif]

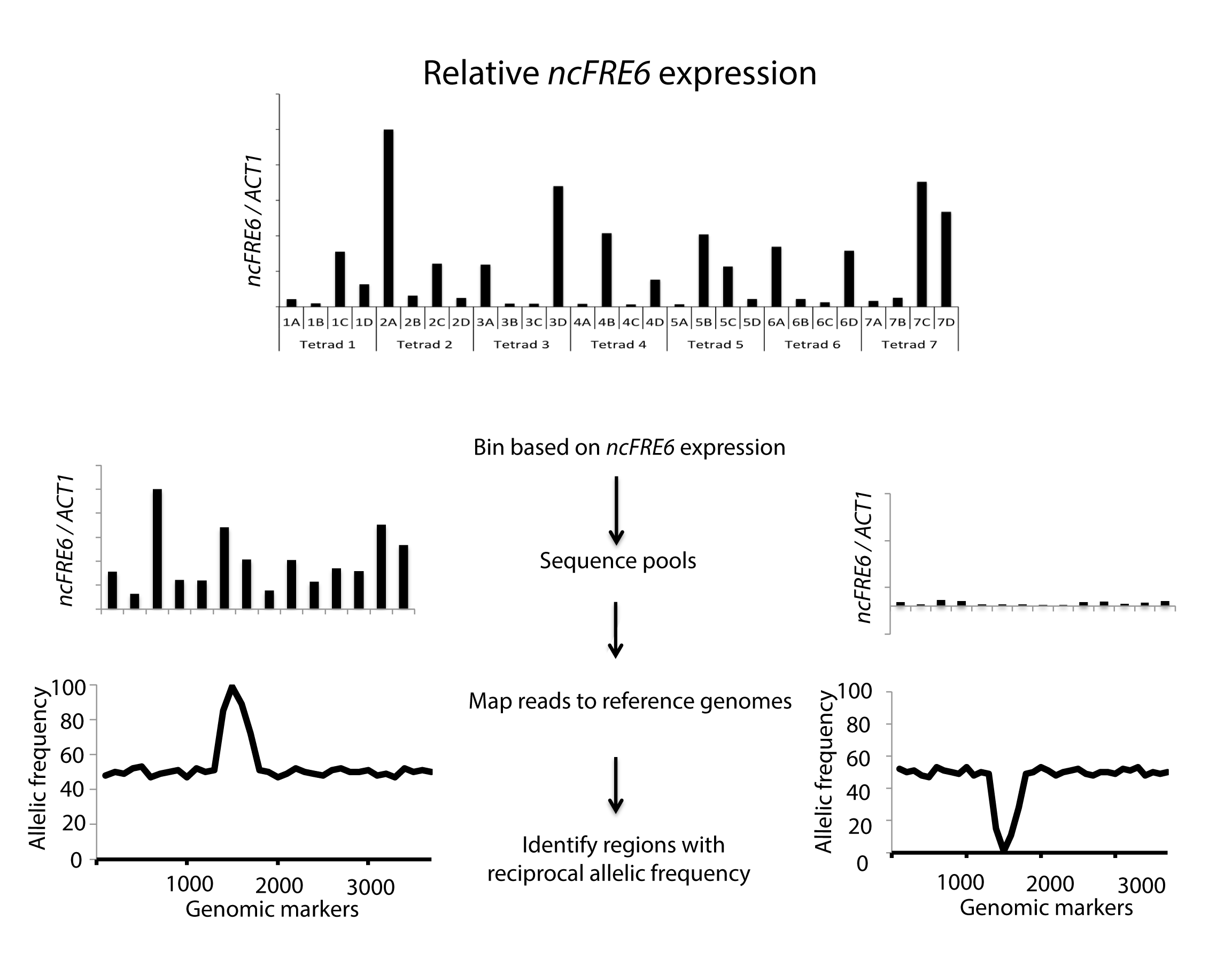

Supplement: S4 Fig — Briefly, segregants were binned based on whether they express AQY2/ncFRE6. Genomic DNA from the expressing group was pooled separately from genomic DNA from the non-expressing pool. Pools were sequenced and reads mapped to both the S288c and ∑1278b genomes. The allelic frequency for each single nucleotide polymorphism is quantified based on read counts mapping to each genome for each pool. A region where only S288c alleles exist in one pool (i.e. expressors) and only ∑1278b alleles exist in the other (i.e. non-expressors) harbor the variant driving differential expression of the transcript being interrogated (i.e. AQY2/ncFRE6). (TIF) [file pgen.1005746.s004.tif]

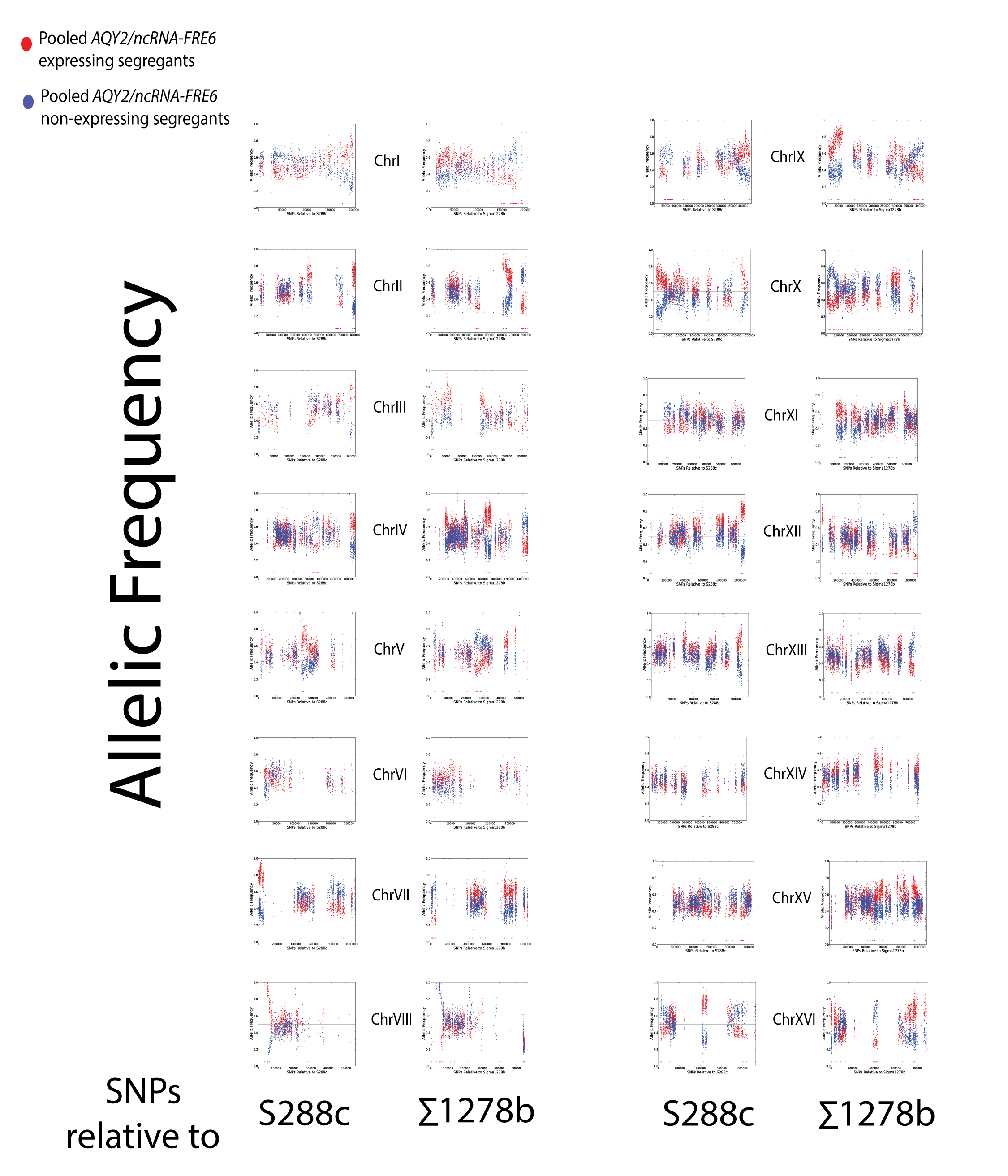

Supplement: S5 Fig — Scatter plots displaying the allelic frequencies of every SNP between S288c and ∑1278b within pools of genomic DNA from either expressing or non-expressing segregants (Red = expressing, Blue = non-expressing). Plots are arranged by chromosome and genome mapped against. X-axis is position along the chromosome. Y-axis is allelic frequency. Red dots represent SNP frequency within the expressing pools. (TIF) [file pgen.1005746.s005.tif]

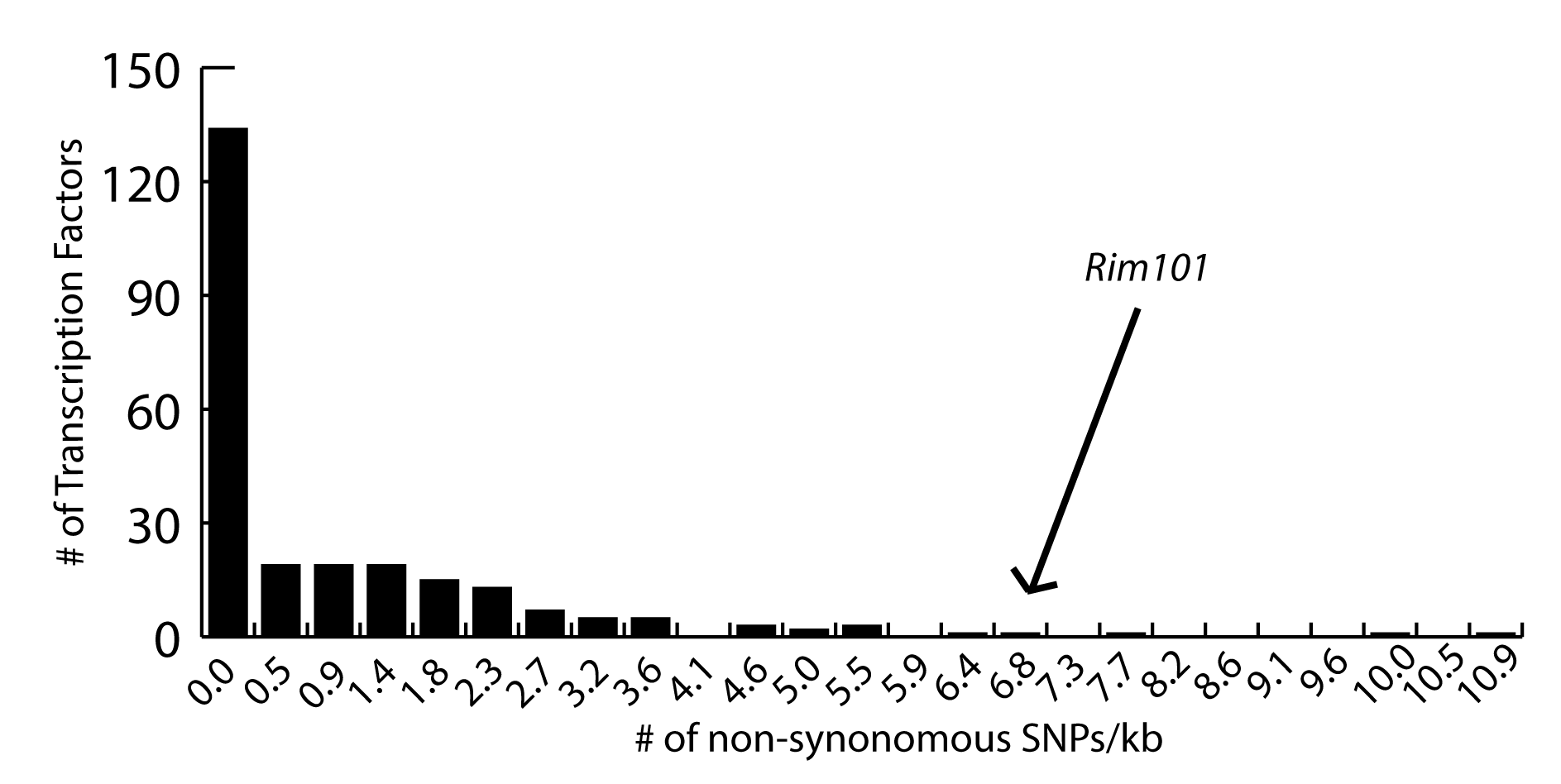

Supplement: S6 Fig — Histogram displays the number of single nucleotide polymorphisms in 249 DNA-binding proteins. X-axis represents number of non-synonomous SNPs/kb. Y-axis is number of transcription factors. (TIF) [file pgen.1005746.s006.tif]

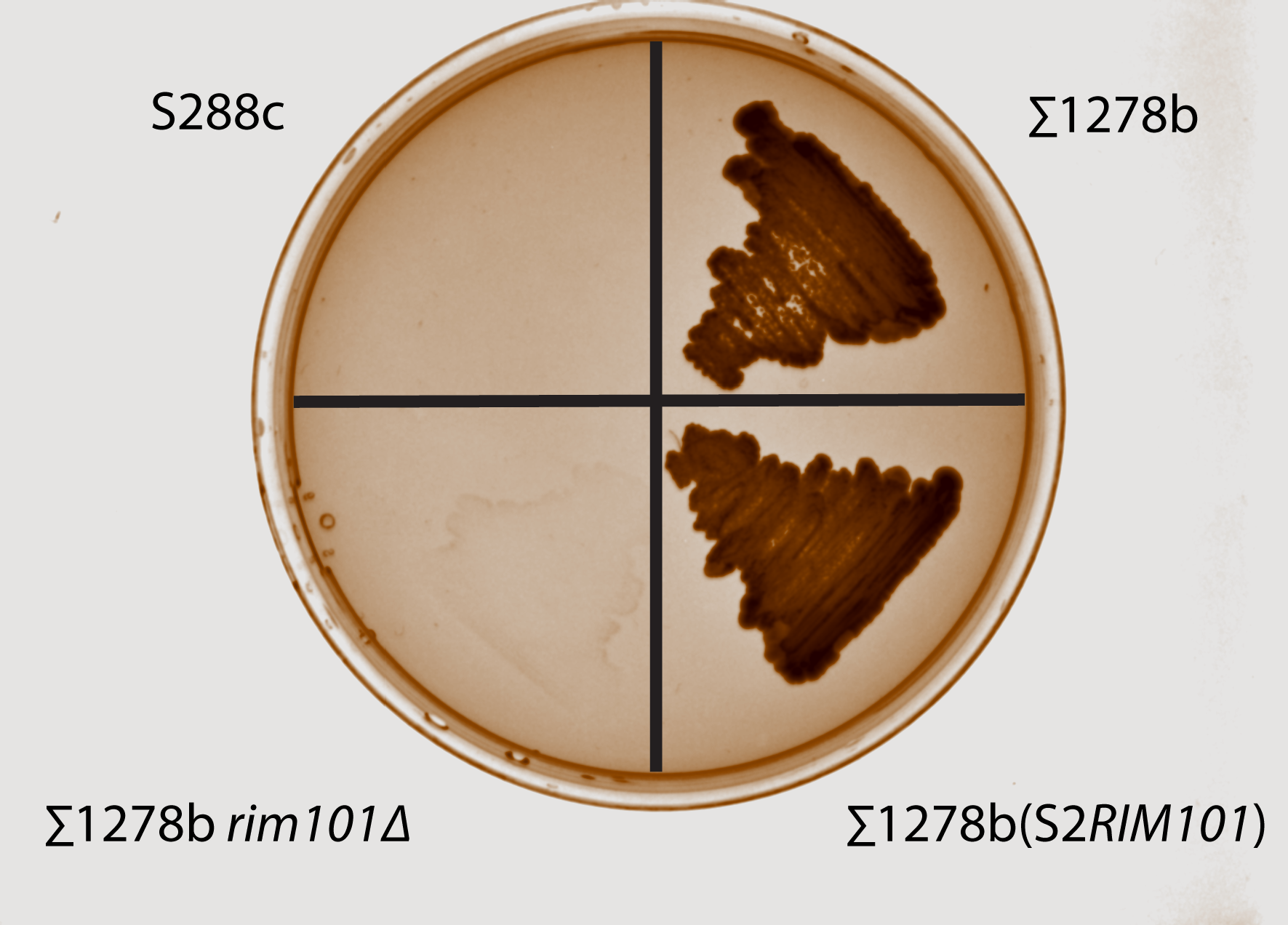

Supplement: S7 Fig — Strains were patched to YPD for two days and washed with gently running water before imaging. (TIF) [file pgen.1005746.s007.tif]

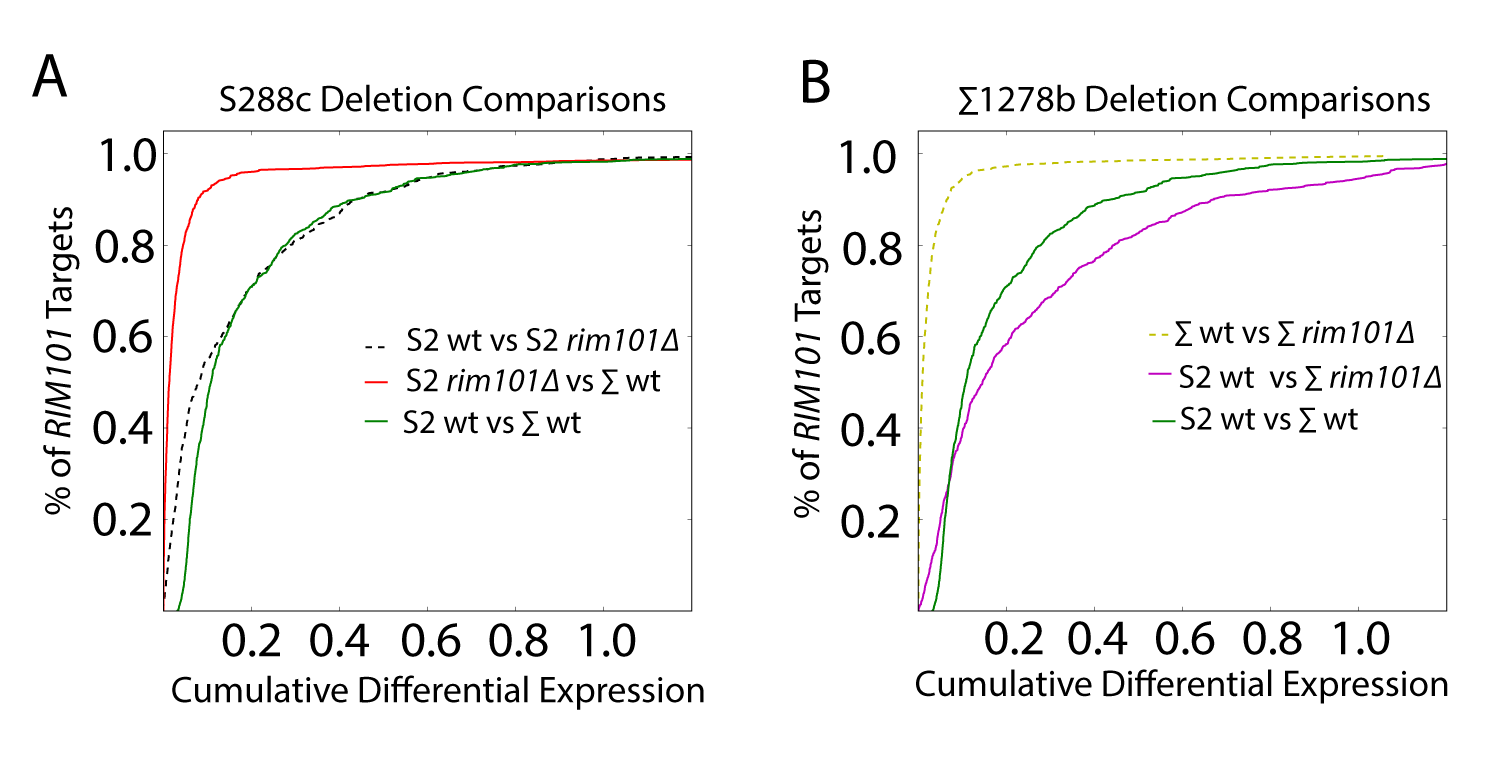

Supplement: S8 Fig — CDF plot examining the impact of deleting RIM101 on 822 RIM101 targets in (A) S288c or (B) ∑1278b. Y-axis represents percentage of RIM101 targets. X-axis represents cumulative differential expression for each comparison. Comparisons specified using S2 (S288c) and ∑ (∑1278b) as abbreviations. (TIF) [file pgen.1005746.s008.tif]

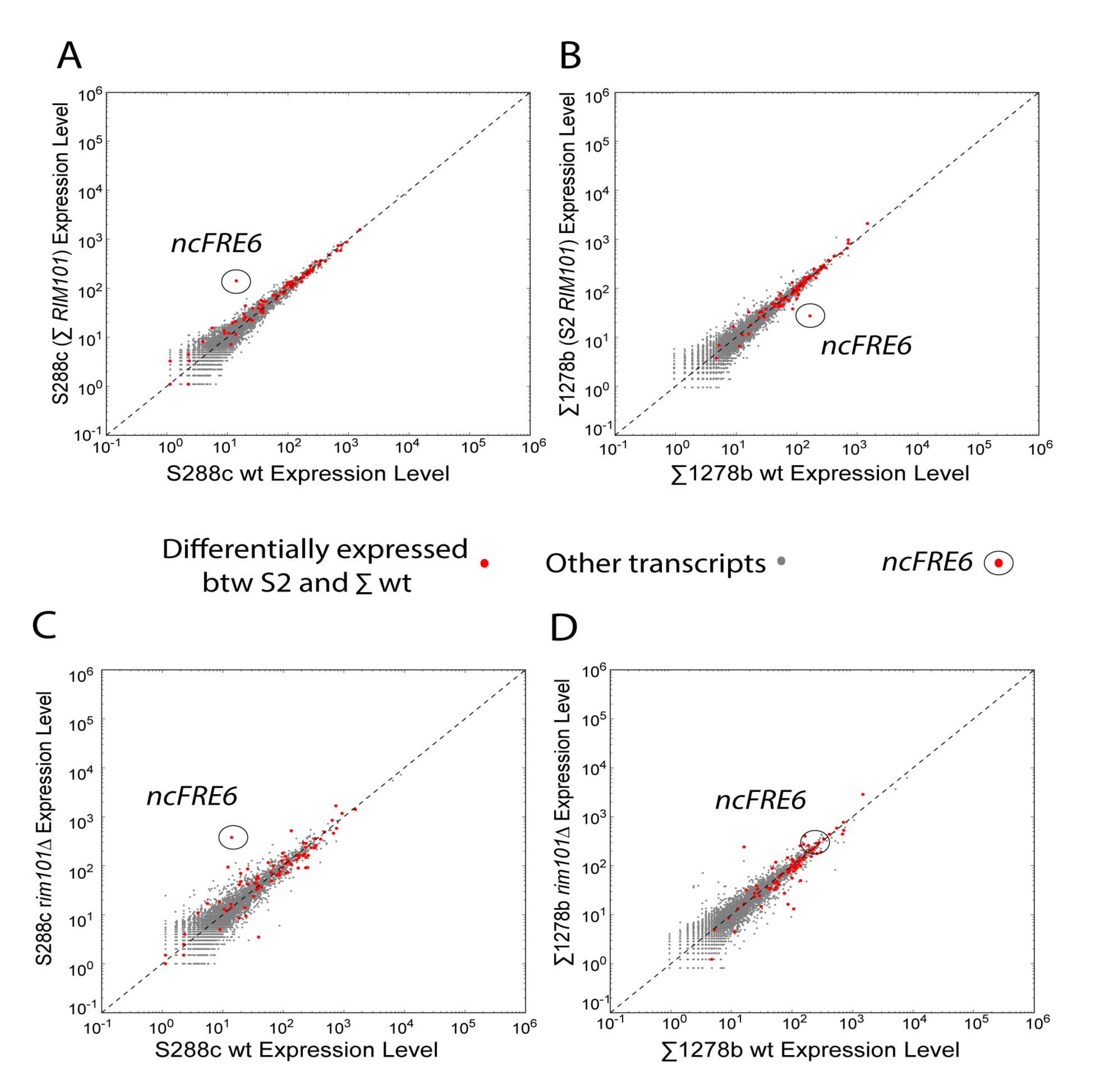

Supplement: S9 Fig — (A) Scatter plot displaying expression levels for each antisense transcript (dots) in S2(∑RIM101) relative to S288c wildtype (Red = RIM101-dependent genes as defined in Fig 4). (B) Scatter plot displaying expression levels for each antisense transcript in ∑1278b(S2RIM101) relative to ∑1278b wildtype. (C) Scatter plot displaying expression levels for each antisense transcript in S288c rim101∆ relative to S288c wildtype. (D) Scatter plot displaying expression levels for each antisense transcript in ∑1278b rim101∆ relative to ∑1278b wildtype. (TIF) [file pgen.1005746.s009.tif]

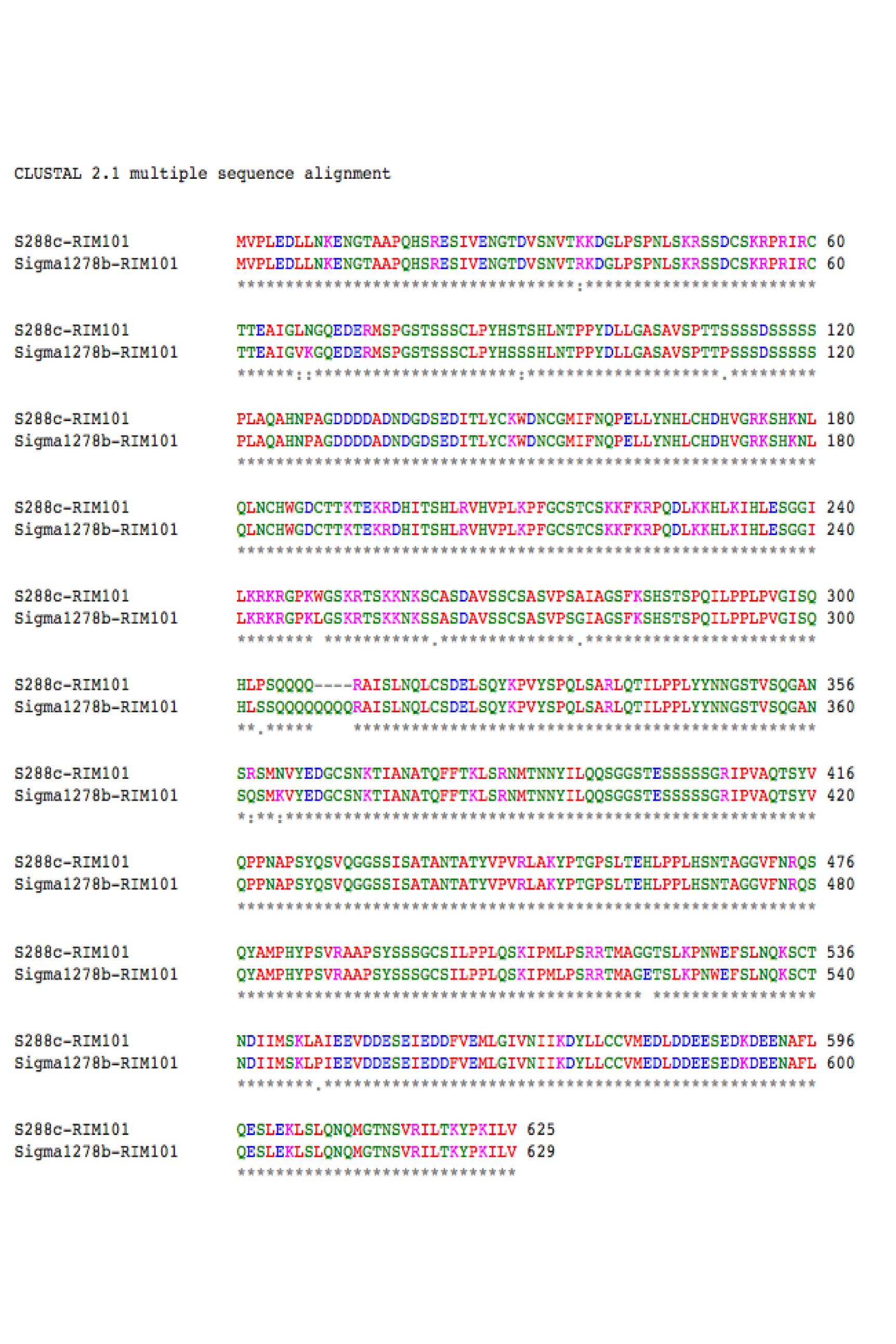

Supplement: S10 Fig — ClustalW protein alignment of Rim101 showing 13 amino acid substitutions and a truncated poly-glutamine tract in S288c relative to ∑1278b. (TIF) [file pgen.1005746.s010.tif]

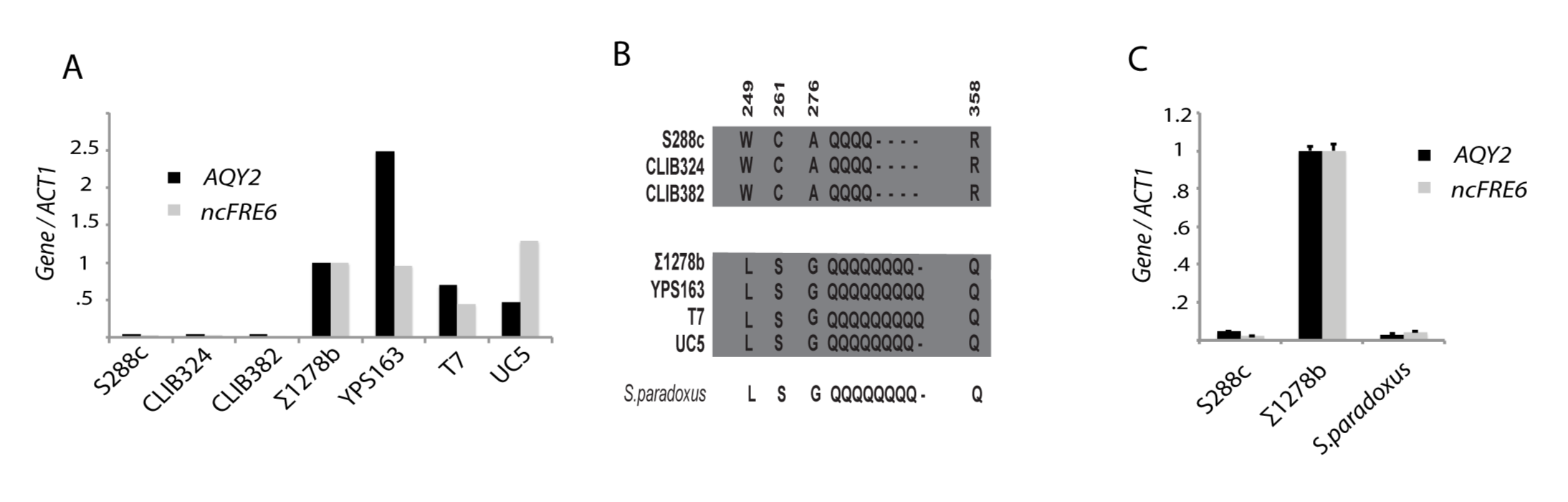

Supplement: S11 Fig — (A) qRT-PCR of AQY2 (Black) and ncFRE6 (grey) in five additional strains of S. cerevisiae. (B) Alignment of the region of Rim101 implicated in repression of AQY2/ncFRE6. (C) qRT-PCR of AQY2 and ncFRE6 in S288c, ∑1278b, and S. paradoxus. (TIF) [file pgen.1005746.s011.tif]
