# Supplementary material for: A trans-acting Variant within the Transcription Factor RIM101 Interacts with Genetic Background to Determine its Regulatory Capacity
Source: PLoS Genet. 2016 Jan 11;12(1):e1005746. doi: 10.1371/journal.pgen.1005746 (PMC4709078; doi:10.1371/journal.pgen.1005746)
Supplement: S1 Text — Extended description of procedures and sequencing statistics. (DOCX) [file pgen.1005746.s017.docx]

**S1 Text: Supplemental Experimental Procedures**

**Chromatin Immunoprecipitation**

Briefly, *Reb1* C-terminal myc tagged strains SAV261 (S288c) and SAV273 (∑1278b) were derived from BY4742 (S288c) and L6441 (∑1278b) and generously provided by Gerald Fink (MIT). 50mL cultures were grown to mid-log phase in YPD at 30^o^C and fixed with 1% formaldehyde for 30 minutes and quenched with glycine for 10 minutes. Cells were pelleted, washed 1X with 10mL 1X TBS, and snap frozen. Cells were lysed in lysis buffer containing 50mM HEPES-KOH pH7.5, 140mM NaCl, 1mM EDTA, 1% Triton X-100, 0.1% sodium deoxycholate, and protease inhibitors by bead beating 5X for 4 minutes at 4^o^C. Lysate was transferred to a 15mL conical, centrifuged at 14000xg, and the pellet was washed 2X with 10mL lysis buffer. Chromatin was sheared using a Diagenode Bioruptor and immunuprecipitation was performed using anti-myc antibody (Sigma cat #: M4439) conjugated to protein G beads (Life technologies cat #: 10004D) overnight at 4^o^C. Beads were washed using a Dynal magnetic stand 2X with lysis buffer, 2X with lysis buffer + 500mM NaCl, and 2X with wash buffer containing 10mM Tris-HCl pH 8.0, 250mM LiCl, .5% NP40, 0.5% sodium deoxycholate, and 1mM EDTA. Chromatin was eluted from beads in TE + 1% SDS for one hour at 65^o^C and cross-links were reversed in TE + 0.5% SDS for 8 hours at 65^o^C. DNA was purified using a Qiagen PCR Purification kit (Qiagen cat #: 28104) and sheared to a median size of 300bp before proceeding to qPCR or library prep. qPCR was performed using primers specified in supplemental materials and methods. Sequencing libraries were prepared by blunting the sheared DNA, A-tailing, and ligating Illumina Tru-seq adapters. Libraries were size selected and purified using a 2% agarose gel and a Qiagen Gel extraction kit. Adapter-ligated libraries were PCR amplified for 18 cycles and sequenced on a 1x50 flow cell on the Illumina HiSeq 2000.

**Identification of disrupted TF binding motifs in the *AQY2/ncFRE6* promoter**

The Yetfasco (1) “Scan sequences” tool to identified all TF binding motifs that exist within the S288c *AQY2/ncFRE6* SNP-dense region requiring a 95% match to a high quality motif. We cross-referenced the list to the list obtained through the same analysis in ∑1278b to obtain a list of motifs that exist in one strain but not the other due to a mutation(s) within the binding motif (Table S3).

**Computational analysis of ChIP-seq data**

**Mapping**

S288c *Reb1* ChIP-seq and ∑1278b *Reb1* ChIP-seq reads were mapped to the S288c reference genome (*S. cerevisiae* genome obtained on 06/26/2011, from from the *Saccharomyces* Genome Database, FTP SITE: <http://downloads.yeastgenome.org/sequence/S288C_reference/genome_releases/> corresponding stable release from February 2011: http://downloads.yeastgenome.org/sequence/S288C_reference/genome_releases/

S288C_reference_genome_R64-1-1_20110203.tgz) using Bowtie2 in very-sensitive end-to-end mode with default score settings (v2.2.3) (2). They were then converted to BAM format using Samtools view, sort, and index (v0.1.18)(3), and duplicate reads were removed via Picard’s MarkDuplicates.jar (1.72). For visualization purposes, the duplicate removed reads were converted to pileup format using Bedtools (v2.16.2) (4) GenomeCoverageBed with the –d option for every position in the genome, and then normalized by read depth(total number of mapped reads).

**Peak Calling and Motif Enrichment**

The peak caller MACS2 (v2.0.9) (5) was run on the non-deduplicated mapped files using broad peaks and allowing for up to 5 duplicate reads at any position, resulting in ~1700 peaks for both S288c and ∑1278b. Those peaks were then subjected to a score cutoff (greater than or equal to 50) and compared (using bedtools intersectBed (v2.16.2) with any overlap). The unique peaks as determined from the intersection were then queried back to the original 1700 peaks for the other strain, to remove artifacts created by the score cutoff. This resulted in a conservative list of strain-unique peaks (68 in S288c and 25 in ∑1278b). Motif enrichment analysis was performed on all peaks with quality score greater than 50 using MEME (v4.10.4) (6) looking for motifs of length 8-10.

**Computational analysis of expression-guided bulked segregant analysis (eBSA)**

**Overall**

The overall method of analysis for identifying the overrepresented alleles in the two pooled expression-guided bulked segregant analysis samples is as follows (Fig S4): First, the two samples were mapped to both the S288c and ∑1278b reference genomes. SNPs were called for each pool relative to either genome, with the expectation that SNPs will be heterozygous (i.e. having relatively equal allelic representation at all locations that did not affect expression of *AQY2/ncFRE6*). SNPs that were called as homozygous in both pools with reciprocal orientation (i.e. homozygous for one allele in the first pool and the other allele in the second pool), that were also called when reads were mapped to the opposite reference genome, were considered potentially linked to expression of *AQY2/ncFRE6*. Only one region of consistent homozygosity (multiple homozygous SNPs in succession) met these criteria: A ~35kb region on chromosome 8 containing 12 genes, including *RIM101* (Fig S5).

**Raw data**

The raw data, single-end 50bp reads, was obtained from the University of Colorado—Denver High Throughput Sequencing Core on the HiSeq2500. The two pooled samples (*AQY2/ncFRE6* expressors versus non-expressors), contained 19.5 and 25.1 million reads each, respectively. Raw reads were tested for adapter read-through or low quality using the FastQC tool (v0.11.2) (7).

**Mapping and variant Calling**

High quality reads, after qualitative inspection from FastQC, from each pooled sample were mapped to each of two *Saccharomyces cerevisiae* reference genomes. The reference sequence for the laboratory yeast strain S288c reference genome (*S. cerevisiae* genome obtained on 06/26/2011, from the *Saccharomyces* Genome Database, FTP SITE: <http://downloads.yeastgenome.org/sequence/S288C_reference/genome_releases/> corresponding stable release from February 2011: http://downloads.yeastgenome.org/sequence/S288C_reference/genome_releases/ S288C_reference_genome_R64-1-1_20110203.tgz) as well as the reference sequence for the laboratory strain ∑1278b (reference genome obtained from Dowell 2010)(8) . Reads were mapped using Bowtie2 in very-sensitive end-to-end mode with default score settings (v2.2.3)(2). After mapping, read information was converted into binary format for downstream analysis using Samtools view, sort, and index (v0.1.18)(3). Variant calling was performed on the tailored read mappings using GATK Unified Genotyper with default settings (v2.4-9)(9). Custom scripts were used to parse out and graph allelic frequencies on a per-SNP basis.

**Identification of the region of the genome harboring RIM101 from Pooled Sequencing**

In order to identify the single locus that segregated with expression of *AQY2/ncFRE6*, we parsed the allelic frequencies of every SNP called in the union of both groups when mapped against each genome. We searched for a genomic region matching the following criteria: 1) a region with high quality variants not representative of mapping artifacts due to differences between the genomes, 2) the region should be homozygous in both groups (i.e. all segregants within the pool had the either the S288c or the ∑1278b allele), 3) the orthologous region should also be homozygous when mapped against the other genome, and 4) the SNPs around the boundaries should gradually decrease in allelic frequency away from a binary homozygous region towards an even 1:1 ratio of alleles in each pooled set. Only one region fit this criteria, a roughly 35Kb region on the left arm of chromosome eight (v2.1.19)(10).

**Analysis of non-synonymous SNPs in transcription factors**

We sought to determine whether RIM101 was more or less polymorphic than all other transcription factors. Briefly, we split the genome of ∑1278b into 150mer reads, mapped them back to S288c using Bowtie2, and called SNPs using GATK Unified Genotyper to identify regions where the two genomes differ. Using custom scripts, we annotated SNPs over coding regions, including whether or not the SNPs cause amino acid changes. An entire list of proteins with annotated DNA binding domains (n=249) was retrieved from YetFasCo (1) and was plotted as a histogram of non-synonymous mutations per kilobase for each gene (Fig S6).

**RNA Sequencing**

**Overall**

Single end, strand-specific RNA-seq was performed on six strains in biological duplicate. In order to discover the differentially expressed genes, the data was mapped back to its respective genome (S288c or ∑1278b), read counts over annotated genes were collected, each gene count was normalized for total depth over non-Ribosomal regions on a per-sample basis, and then the genes that exist in both genomes had their expression levels compared to identify those genes that are significantly different in expression.

**Raw data**

Raw data consisted of single-end 126bp reads, obtained from University of Colorado—Denver High Throughput Sequencing Core and was sequenced on the HiSeq2500. Raw reads were first converted into their reverse complement (due to the NEBNext Ultra-sensitive strand specific library prep) and sent through quality analysis to identify possible adapter read-through or quality-score biases using the FastQC tool (v0.11.2)(7). We observed a large amount of adapter in the 3’ end of reads. Hence, we hard-trimmed the reads to 50bp in the mapping process (below).

**Mapping**

Reads were mapped to their respective genomes (see Genome Sequencing) using Bowtie2 (2). We trimmed the reads to 50bp (bowtie2 option -5 76). We ran bowtie2 with --very-sensitive end-to-end alignment, and adjusted our scoring scheme to limit mismatches and insertions and deletions (-L,-20,0). They then underwent file format conversion into the binary format for downstream analysis using Samtools view, sort, and index (v0.1.18) (3). Read mapping statistics are as follows:

| Sample | Total Reads | Total Mapped | Replicate Pearson Correlation Coefficient |
| --- | --- | --- | --- |
| S288c_wt_rep1 | 20629107 | 19494132 | .99622 |
| S288c_wt_rep2 | 23279566 | 22241295 | .99622 |
| S288c_∑RIM101_rep1 | 21984130 | 20689010 | .99832 |
| S288c_∑RIM101_rep2 | 20697838 | 19804140 | .99832 |
| S288c_RIM101deleted_rep1 | 22891460 | 21029594 | .99687 |
| S288c_RIM101deleted_rep2 | 22651091 | 20403109 | .99687 |
| Sigma_wt_rep1 | 22893870 | 21484712 | .99433 |
| Sigma_wt_rep2 | 21994461 | 20801472 | .99433 |
| Sigma_S2RIM101_rep1 | 24951411 | 23444650 | .98974 |
| Sigma_S2RIM101_rep2 | 20918844 | 19767717 | .98974 |
| Sigma_RIM101deleted_rep1 | 21961295 | 20384668 | .99485 |
| Sigma_RIM101deleted_rep2 | 23072053 | 21499624 | .99485 |

**Quantification and Differential Expression**

Per-gene read counts were attained using HTSeq over the coding regions present in the annotations for each genome (v0.6.1)(11). After acquiring read counts in each genome, the genes that exist in both genomes were placed into a count matrix (gene x sample), dubious ORFs were removed, Pearson correlation coefficients were calculated (table above), +1 smoothing was added to every gene (to remove divide-by-zero errors) and read into the R package for differential expression DESeq (v1.0)(12). The output of the DESeq analysis identified differentially expressed genes with an adjusted p-value, as well as a Log2 fold change for the comparison. For stringency, a cutoff for differential expression of p-adj < 0.0005 and a minimum average expression between the comparisons of ≥ 100 reads was used. We observed roughly ~20% of genes as differentially expressed at this stringent cutoff. Since the data maintained strand information, HTSeq was run separately on antisense transcripts for each gene. The antisense gene counts were processed as above, but with a lower number of required reads (50 vs. 100) mapped, because antisense transcripts typically show lower expression than sense transcripts.

**Statistical Analysis of Cumulative Differential Expression**

In order to quantify the “cumulative differential expression”—or measure of the total difference between two samples’ expression values—for a set of genes we used the residual sum of squares (RSS) in log-space for a set of genes relative to the linear regression fit to the background set of genes (entire set minus the gene set in question). We plot the RSS for individual comparisons as a CDF to highlight a reduction in the distribution of cumulative differential expression for different pairwise comparisons. In order to assess the significance of reduction of cumulative differential expression, we use the ANOVA one-tailed F-test to evaluate whether the variance between pairwise comparisons was equal (H_0_: VarA = VarB), or whether the variance was lower (H_I_: VarA < VarB).

**Plasmids used in this study**

The pCORE plasmid was constructed by cloning a 1.5 kb *Bam*HI-*Hin*cII fragment harboring the *kanMX4* gene into the *Bam*HI-*Ssp*I sites of pFA6aKlURA3 (Storici et al. 2001).

**Primers used in this study**

**Primers used to generate Reb1 binding motif mutants:**

For amplification of pCORE to replace Reb1 SNP in both backgrounds:

Forward primer:

ACCAACACTGATATTCCTCGAAATACTCTATAATTCTCTCGAGCTCGTTTTCGACACTGG

Reverse primer:

TGTTAGAAACACCGTTTCTCAAAAACTCCTCGGTTACCCTCCTTACCATTAAGTTGATC

Primers to amplify genomic sequence of S288c or ∑1278b for replacement of Reb1 binding site SNP:

Forward primer:

GAAGGAGCCGGAGAGAAGAT

Reverse primer:

GGAGATTCATTAGCGGTCGT

Primers to test Reb1 occupancy by ChIP-qPCR:

Forward primer:

GGAGATTCATTAGCGGTCGT

Reverse primer:

GAAGGAGCCGGAGAGAAGAT

**Primers used to generate *AQY2/ncFRE6* *cis* context mutants:**

For amplification of pCORE to replace 30 SNPs (i.e. S288c(30 ∑ SNPs) or ∑1278b(30 S2 SNPs)):

Forward primer:

CGGCTGTTCAGGTGGAATATAAGCATTGTCAACACCGGTGAGCTCGTTTTCGACACTGG

Reverse primer:

TTGTTGGCAACACGTCAAAATTTTCAACGGTTGGAAAGATCCTTACCATTAAGTTGATC

Primers for amplification of genomic DNA from S288c or ∑1278b to create template for transformation and counter selection. Includes 30 SNPs within the *AQY2/ncFRE6* *cis* context:

Forward primer

TGGAATATAAGCATTGTCAACACC

Reverse primer

GCCCTTTTGTTCTTTTACTGTTG

For amplification of pCORE to replace 15 *AQY2* proximal SNPs in ∑1278b (i.e. ∑1278b(15 S2 SNPs)):

Forward primer:

AGGAACAAGAAAAAAGACATGCGCACACTAATAAGCTACGAGCTCGTTTTCGACACTGG

Reverse primer:

GGAGGTGGCGCTGCAGTCCTTCTTTTCAGACCCAAGCAATCCTTACCATTAAGTTGATC

For this strain a gBLOCK fragment (Integrated DNA Technologies) was synthesized to replace all 15 SNPs in ∑1278b with those from S288c.

**Primers used to generate *RIM101* mutant strains:**

Primers for amplification of pCORE for targeting to S288c *RIM101* ORF (S288c *rim101∆* strains used in the study).

Forward primer

ACTGAAAACGGTAAAGTAGGTTTGTTTAAATTGACTTAAGGAGCTCGTTTTCGACACTGG

Reverse primer

GCAAAGAAACAACTAAGAATAAAATATCCGACAATCCATATCCTTACCATTAAGTTGATC

To amplify pCORE for targeting to ∑1278b *RIM101* ORF (∑1278b *rim101∆* strain used in this study).

Forward primer

ACTGAAAACGGTAAAGTAAGTTTGTTTAAATTGACTTAAGGAGCTCGTTTTCGACACTGG

Reverse primer

GCAAAGAAACAACTAAGAATAATATATCCAACAATTCATATCCTTACCATTAAGTTGATC

Primers for interconversion of *RIM101* allele between strains (after insertion of pCORE in place of RIM101).

Primers for amplification of *RIM101* allele from S288c for transformation into ∑1278b *rim101∆*:

Forward primer

AACAAGTGCAAAGATAAAATACTGAAAACGGTAAAGTAAGTTTGTTTAAATTGACTTAAG

Reverse primer

TACTATACAGCCGCAAAGAAACAACTAAGAATAATATATCCGACAATTCATATCATACCA

Primers for amplification of ∑1278b *RIM101* allele for transformation into S288c *rim101∆*

Forward primer

AACAAGTGCAAAGATAAAATACTGAAAACGGTAAAGTAGGTTTGTTTAAATTGACTTAAG

Reverse primer

TACTATACAGCCGCAAAGAAACAACTAAGAATAAAATATCCAACAATCCATATCATACCA

Primers for interconverting PolyQ repeat lengths between S288c and ∑1278b

To amplify pcore for replacement of S288c polyQ repeat:

Forward primer

CCCCCATTGCCCGTGGGTATATCTCAACATCTGCCTTCAGAGCTCGTTTTCGACACTGG

Reverse primer

TAGCTCGTCTGAGCATAGTTGGTTTAAGGAAATAGCCCGTCCTTACCATTAAGTTGATC

Primers to amplify pcore for targeting to ∑1278b polyQ repeat:

Forward primer

CCCCCATTGCCCGTGGGTATATCTCAACATCTGTCTTCAGAGCTCGTTTTCGACACTGG

Reverse primer

TAGCTCGTCTGAGCATAGTTGGTTTAAGGAAATAGCCCGTCCTTACCATTAAGTTGATC

Primers to amplify pCORE for replacement of four individual amino acid residues implicated in regulation of *AQY2/ncFRE6*.

Forward primer

GAAAGTGGCGGTATTTTGAAAAGAAAGAGGGGACCCAAATGAGCTCGTTTTCGACACTGG

Reverse primer

CGTTTGCTATGGTCTTATTAGAACAACCGTCCTCGTAGACTCCTTACCATTAAGTTGATC

Once pCORE inserted, primers used to check incorporation:

Forward primer

TCATCTGGAAAGTGGCGGTA

Reverse primer

GTGAAGAATTGGGTGGCGTT

gBLOCKs (IDT) were synthesized with each SNP, transformed, and counter selected for loss of the pCORE construct.

*All strains were confirmed by PCR and Sanger sequencing. Strains were initially selected for incorporation of the pCORE construct by growth on G418 and SC –ura and lack of growth on 5-FOA. After replacing the pCORE construct strains were tested for growth on 5-FOA and YPG and no growth on G418 or SC –URA. For polyQ repeat length strains and individual amino acid substitution strains, gBLOCK fragments were synthesized to incorporate altered polyQ lengths.

**Primers used for qRT-PCR experiments:**

Primer for gene-specific reverse transcription of *ncFRE6*:

CAGTGCTTTGCGTTCTACTA

**qPCR primers:**

Primers to measure *ncFRE6*

Forward primer

ATCGCTCGGAATAGTAAGGAAA

Reverse primer

CCCCAAATGAGCAAGGATAC

Primers to measure *ncFRE6* in Figures 4A, Figure S8.

Forward primer

TTTGAACACCAGCAACAACC

Reverse primer

ACAATATTGACCCGGTTTCG

Primers used to measure *AQY2*:

Forward primer

AACAGCCTAAACCCAAAGCA

Reverse primer

GCCGCTAGTGCTATGACTCC

Primer used for RT of ncFRE6 in *S.paradoxus*:

GGATCGTGCTGTCCTTGTTC

Primers to detect ncFRE6 in *S.paradoxus*:

Forward primer:

TTGAACACCAGCAACAACCC

Reverse primer:

GTCCCGGTTTTGAATGCCAT

Primers to detect *AQY2* in *S.paradoxus*:

Forward primer:

ACATTTCACACCTGGACCCA

Reverse primer:

TTTGTTTCCGGCTGTTCAGG

**Primers used to detect relative occupancy at the *Reb1* SNP located near the start of *ncFRE6* by ChIP-qPCR:**

Forward primer:

GGAGATTCATTAGCGGTCGT

Reverse primer:

GAAGGAGCCGGAGAGAAGAT

**Supplemental references**

1. De Boer CG, Hughes TR. YeTFaSCo: A database of evaluated yeast transcription factor sequence specificities. Nucleic Acids Res. 2012;40(D1):169–79.

2. Langmead B, Salzberg SL. Fast gapped-read alignment with Bowtie 2. Nat Methods. 2012;9(4):357–9.

3. Li H, Handsaker B, Wysoker A, Fennell T, Ruan J, Homer N, et al. The Sequence Alignment/Map format and SAMtoolsLi, H., Handsaker, B., Wysoker, A., Fennell, T., Ruan, J., Homer, N., … Durbin, R. (2009). The Sequence Alignment/Map format and SAMtools. Bioinformatics, 25(16), 2078–2079. doi:10.1093/bioinformatics/btp352. Bioinformatics. 2009;25(16):2078–9.

4. Quinlan AR, Hall IM. BEDTools: a flexible suite of utilities for comparing genomic features. Bioinformatics [Internet]. 2010;26(6):841–2. Available from: http://www.pubmedcentral.nih.gov/articlerender.fcgi?artid=2832824&tool=pmcentrez&rendertype=abstract\nhttp://bioinformatics.oxfordjournals.org/content/26/6/841.short

5. Zhang Y, Liu T, Meyer C a, Eeckhoute J, Johnson DS, Bernstein BE, et al. Model-based Analysis of ChIP-Seq (MACS). Genome Biol [Internet]. 2008;9(9):R137. Available from: http://genomebiology.com/2008/9/9/R137

6. Bailey TL, Boden M, Buske F a., Frith M, Grant CE, Clementi L, et al. MEME Suite: Tools for motif discovery and searching. Nucleic Acids Res. 2009;37(SUPPL. 2):202–8.

7. Leggett RM, Ramirez-Gonzalez RH, Clavijo BJ, Waite D, Davey RP. Sequencing quality assessment tools to enable data-driven informatics for high throughput genomics. Front Genet. 2013;4(DEC):1–5.

8. Dowell RD, Ryan O, Jansen A, Cheung D, Agarwala S, Danford T, et al. Genotype to Phenotype : A Complex Problem. 2010;(April):2010.

9. McKenna, A et al. The Genome Analysis Toolkit: A MapReduce framework for analyzing next-generation DNA sequencing data. Genome Res. 2010; 20:1297-1303

10. Thorvaldsdóttir H, Robinson JT, Mesirov JP. Integrative Genomics Viewer (IGV): High-performance genomics data visualization and exploration. Brief Bioinform. 2013;14(2):178–92.

11. Anders S, Pyl PT, Huber W. HTSeq A Python framework to work with high-throughput sequencing data. bioRxiv. 2014;31(2):002824.

12. Anders S, Huber W. Differential expression analysis for sequence count data. Genome Biol. BioMed Central Ltd; 2010;11(10):R106.
